# Geographic variation in the intended choice of adjuvant treatments for women diagnosed with screen-detected breast cancer in Queensland

**DOI:** 10.1186/s12889-015-2527-2

**Published:** 2015-12-02

**Authors:** Jeff Ching-Fu Hsieh, Susanna M. Cramb, James M. McGree, Nathan A. M. Dunn, Peter D. Baade, Kerrie L. Mengersen

**Affiliations:** Mathematical Sciences, Queensland University of Technology, Brisbane, Queensland, Australia; Cancer Research Centre, Cancer Council Queensland, Brisbane, Queensland, Australia; Preventive Health Unit, Department of Health, Brisbane, Queensland, Australia

**Keywords:** Adjuvant therapies, Bayesian shared spatial-component model, Breast cancer, Mammography screening, Spatial inequalities

## Abstract

**Background:**

Although early diagnosis and improved treatment can reduce breast cancer mortality, there still appears to be a geographic differential in patient outcomes. This study aims to determine and quantify spatial inequalities in intended adjuvant (radio-, chemo- and hormonal) therapy usage among women with screen-detected breast cancer in Queensland, Australia.

**Methods:**

Linked population-based datasets from BreastScreen Queensland and the Queensland Cancer Registry during 1997−2008 for women aged 40−89 years were used. We adopted a Bayesian shared spatial component model to evaluate the relative intended use of each adjuvant therapy across 478 areas as well as common spatial patterns between treatments.

**Results:**

Women living closer to a cancer treatment facility were more likely to intend to use adjuvant therapy. This was particularly marked for radiotherapy when travel time to the closest radiation facility was 4 + h (OR =0.41, 95 % CrI: [0.23, 0.74]) compared to <1 h. The shared spatial effect increased towards the centres with concentrations of radiotherapy facilities, in north-east (Townsville) and south-east (Brisbane) regions of Queensland. Moreover, the presence of residual shared spatial effects indicates that there are other unmeasured geographical barriers influencing women’s treatment choices.

**Conclusions:**

This highlights the need to identify the additional barriers that impact on treatment intentions among women diagnosed with screen-detected breast cancer, particularly for those women living further away from cancer treatment centers.

**Electronic supplementary material:**

The online version of this article (doi:10.1186/s12889-015-2527-2) contains supplementary material, which is available to authorized users.

## Background

Breast cancer is the most commonly diagnosed cancer among Australian women and the second most common cause of cancer-related death for Australian females [1, 2]. Improved survival outcomes over time among women diagnosed with breast cancer have been observed across the developed world, due to a combination of earlier diagnosis through mammography screening along with improved treatment [1–6]. However, patient care pathways can vary substantially to those recommended by clinical practice guidelines [7–11].

Typically, breast cancer treatment involves surgery, with the option of one or more types of adjuvant therapy. Adjuvant therapy is additional treatment that is commonly given before or after surgery for breast cancer and is designed to improve disease-specific symptoms and overall survival. Adjuvant therapy includes radiotherapy, which is targeted at specific tissue/s, or chemotherapy and hormonal therapy which are systemic treatments that impact on the whole body.

Clinical practice guidelines for the treatment of breast cancer depend on a number of factors including histological type, tumour stage, age of the patient, the informed decisions of the medical staff and the personal decisions of the patient herself [8, 12, 13]. These last two components mean that even if two women have the same clinical and demographic characteristics, their final treatment strategy may be different.

One potential measure of distinction between women is where they live, and hence the relative access to different types of adjuvant treatment. In Queensland, until 2011, radiotherapy was only available in the south-east corner, where the majority of the population lives, and Townsville in the north-east. Since radiation treatment requires daily administration across consecutive weeks, longer distances to these centers can form a utilization barrier. In contrast, chemotherapy can be administrated at more locations, including general practices and hospital outpatient facilities, while hormonal therapy can be administrated via ongoing self-administered oral medication or one off surgical treatment.

A number of international studies have demonstrated that the use of various types of adjuvant therapies varies by rural location [7, 14, 15], age [16], race [17, 18] and access to services [18, 19]. However what is not understood is how these variables impact on women’s selection of different types of adjuvant treatment, and indeed whether a woman chooses to have any adjuvant therapy at all. Indeed, there may be some common spatially-structured underlying factors that influence a woman’s decision to have adjuvant therapy that represents possible unmeasured influences including travel or financial burden and stress.

Following this hypothesis, we can use shared component models within a Bayesian framework [20, 21] to quantify and examine the spatial variations of the unmeasured shared component across the state. The Bayesian shared component model has been shown to be a useful and valuable extension over individual analysis [22] in a spatial setting. Because information is borrowed between responses, the model is able to provide more statistically robust estimates of spatial inequalities in the choice of adjuvant therapies, even when numbers of people diagnosed in specific geographical area are small. Using the clinical and recommended treatment data from the publicly funded and population-based BreastScreen Queensland (BSQ) in Australia, a Bayesian shared spatial component model [20–22] was adopted for the multiple treatment responses. The aim was to identify common (and treatment-specific) spatial patterns across geographic areas and patients’ demographic and clinical characteristics in the intended use of adjuvant therapies (both separately and in combination) for women with screen-detected breast cancer.

## Methods

### Study cohort

The state of Queensland in Australia hosts more than four million people in an area of nearly two million square kilometres, spreading from the populous southeast corner and coastal areas to remote outback regions. The study cohort was obtained by linking data from BSQ and the Queensland Cancer Registry (QCR). This includes women who were diagnosed with invasive breast cancer by BSQ mammography screening from 1 January 1997 to 31 December 2007 and followed-up to 31 December 2008. While specific measurements of data quality for BSQ and QCR data are not available, both data collections have systematic validation processes in place to ensure that the collection and recording of information is as accurate as possible.

BSQ is the only population-based public health breast cancer screening service in Queensland that provides free 2-yearly screening mammograms to women aged 40 and over. BSQ is part of the BreastScreen Australia Program established in 1991 by the Australian Government and the State and Territory governments. In 2007, over 202,000 women from all age were screened by BSQ with a participation rate for the 50−69 target age group of 56 % over the two year period 2006−2007 [1]. Approximately 29 % of invasive breast cancers were diagnosed by screening throughout the study period 1997−2007.

Data linkage was undertaken by BSQ staff using a deterministic matching process with over 90 % matching completeness. Ethics approval was granted by the Human Research Ethics Committee of Queensland University of Technology (approval number: 1100000036). Access to the data was provided by Queensland Health under the Public Health Act 2005 (RD003676). Since only de-identified data was provided by the data custodians and subsequently used in these analyses, no patient consent was obtained.

As treatment information was only available for breast cancers diagnosed by screening, the analysis included only BSQ mammographic screen detected invasive breast cancer (ICD-O-3 code = C50) for women diagnosed at ages 40 to 89 years with information about the intended treatment strategy agreed on by the patient and doctor. There was no specific exclusion criteria in relation to women diagnosed with multiple invasive breast cancers, of whom there were less than 0.1 % in the cohort. Cases were excluded when age at diagnosis, geographic location or treatment information was missing, if cancer was identified at autopsy or by death certificate only, or if subjects had a survival time of less than one day.

### Data description

The primary outcome variable included in the analysis was the intended use of breast cancer adjuvant therapy, being one or more of radiotherapy, chemotherapy and hormonal therapy. No information was available regarding treatment uptake or completion. The information about intended treatment is routinely collected within BSQ, unlike most other BreastScreen services in other Australian states. For patients who chose to be treated in a public facility, the treatment information was collected by BSQ staff via access to the decisions and recommendations made at multi-disciplinary team (MDT) meetings through Queensland Oncology Online (QOOL) or by consulting breast-care nurses in the relevant departments. For BSQ patients who elected to be treated at a private treatment facility, a request was made from the treating surgeon for the intended adjuvant therapy details.

A total of 6,357 women diagnosed with screen-detected invasive cancers with information about the intended treatment procedure (including women who chose to not have any treatment) were included in the study. Of these women, 5,251 of them intended to receive at least one type of adjuvant therapy and more than a third (37.9 %) intended to use both radiotherapy and hormonal therapy, making this the most common choice (Table 1). A further 11.6 % (*n* = 607) intended to use all 3 adjuvant therapies.

**Table 1:** The count and percentage for the adjuvant therapy combination among the study cohort

Type of treatment	N^a^ (pct)
Radiotherapy only	800 (15.241 %)
Chemotherapy only	205 (3.90 %)
Hormonal therapy only	899 (17.12 %)
Radiotherapy and chemotherapy	511 (9.73 %)
Radiotherapy and hormonal therapy	1990 (37.90 %)
Chemotherapy and hormonal therapy	239 (4.55 %)
All three therapies	607 (11.56 %)

^a^Number of patients

Demographic variables extracted were age group at diagnosis (40−49, 50−59, 60−69 and 70−89 years), Indigenous status (Indigenous, non-Indigenous and unknown), marital status at time of diagnosis (married, never married, widowed/divorced/separated or unknown) and occupation (blue collar, white collar, professional, not in the labour force and unknown). Clinical variables were tumour stage at diagnosis (localised, advanced or unknown), type of invasive tumour (invasive ductal, tubular, lobular classical, other and unknown), whether the tumour was diagnosed at the woman’s first mammographic screening episode (yes/no), and intended surgical procedure, irrespective of adjuvant therapy, (classified into breast-conserving surgery, mastectomy, no surgery or unknown).

Patients’ demographic and geographic information, along with tumour stage, were sourced from QCR, while all other clinical information, intended treatment, and screening information were sourced from BSQ. Both marital status and occupation are sourced through the QCR via hospital notifications and death certificates. No additional data cleaning or verification for these variables is conducted within the QCR.

Geographic location information was based on the 2006 version of the Australian Standard Geographical Classification using Statistical Local Areas (SLAs), of which 478 cover Queensland without gap or overlap. SLAs are spatial entities that are deemed to be relatively homogeneous in terms of the socio-economic characteristics of the populations they contain. SLAs are often based on the incorporated bodies of local governments and councils, which are responsible for infrastructure and service provision at the local and regional level. Each SLA was classified according to socio-economic status (SES) as measured by the Index of Relative Socio-economic Advantage and Disadvantage (IRSAD) [23]. This index was categorized into quintiles, with 1 representing most disadvantaged to 5 being most advantaged. A cancer-specific remoteness index was used (TRAvel to Cancer Treatment (TRACT)) which measured the road travelling time between each women’s residential SLA to the closest radiation facility in 2006 using Geographical Information System software and a street network database [24]. The TRACT was categorised into <1, 1- <2, 2- <4, 4- <6 and 6 or more hours. While there was some change in this measure over the study period reflecting new facilities being commissioned, it was decided to base distances on the 2006 data for consistency with the area-level SES and to reduce model complexity. Sensitivity analyses (not published) using different year selections showed little impact on the final results.

**Fig. 1:**
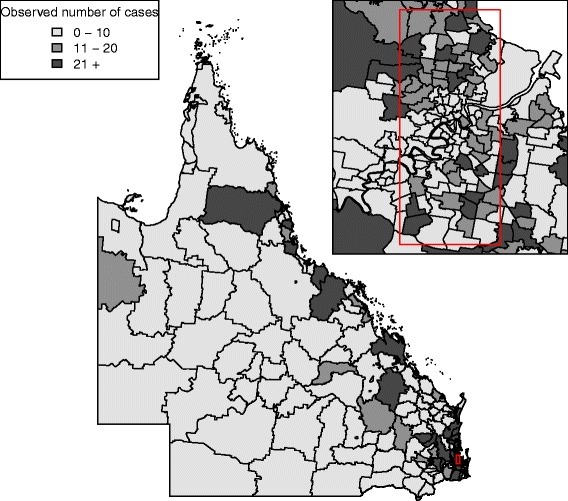
Observed number of screen-detected invasive breast cancer at each SLA across QLD, 1997–2007

The observed number of screen-detected invasive cases was mapped to the 2006 SLA boundaries based on suburb and postcode of residence (see Fig. 1). There are 61 SLAs without patients.

### Statistical model

For a Bayesian shared spatial component model, suppose *Y*_*ijk*_ is the indicator (0/1) of treatment *k*, *k*=1 (radiotherapy), 2 (chemotherapy), 3 (hormonal therapy) for woman *j* in SLA *i*, *i*=1,2,…,478, that follows a Bernoulli distribution, as:

(1) $$ Y_{ijk} \sim Bernoulli(p_{ijk}),  $$

where *p*_*ijk*_ is the probability of the *j*^*th*^ women in SLA *i* having treatment *k* and is described with a logistic regression model as:

(2) $$ logit(p_{ijk}) = \alpha_{k}+\beta_{k}\mathbf{X_{ij}}+u_{ik}+\eta_{ik},  $$

where *α*_*k*_ is the treatment-specific intercept; *β*_*k*_ is the vector of regression coefficients for each treatment (*k*) corresponding to the predictor variables *X*_*ij*_; and *u*_*ik*_ and *η*_*ik*_ represent spatially unstructured and structured random effects respectively, for the *i*^*th*^ SLA and treatment *k*.

In this shared component model, the spatially structured random effects (*η*_*ik*_) are partitioned into components which are shared and specific for each treatment to identify spatial patterns in the residual variation as:

$$\begin{array}{*{20}l} \eta_{i1} & = \delta_{1}\phi_{i}+s_{i1}\\  \eta_{i2} & = \delta_{2}\phi_{i}+s_{i2}\\  \eta_{i3} & = \delta_{3}\phi_{i}+s_{i3}, \end{array} $$

where the terms *δ*_*k*_ are the weight, or scaling parameters, that quantify the relative contribution of the common spatial component *ϕ*_*i*_ in each treatment [25]; *ϕ*_*i*_ the shared spatial component is a latent variable with spatial structure that is shared among all adjuvant therapies and *s*_*ik*_ are the treatment-specific spatial components. To ensure identifiability, a standard constraint of requiring the product of *δ*_*k*_ to be equal to 1 is applied [25, 26].

The prior distribution for the intercepts *α*_*k*_ and regression coefficients *β*_*k*_ were assigned a zero mean Gaussian distribution with a Gamma (0.005,0.5) hyperprior distribution, parameterized in terms of the shape and inverse scale parameters, for the precision (=1/variance). These flat prior distributions were chosen with no knowledge about the relationship between the response and predictors. The unstructured random effects *u*_*ik*_ were described by a multivariate Gaussian prior distribution [21] with a precision matrix *Σ*∼Wishart(*Q*,5) where *Q* is a 3×3 identity matrix to allow for correlation among the treatments. The logarithms of the weights were assigned Gaussian (0,5.9) (precision =5.9) prior distributions [27], and following the Besag et al. [28] framework the shared and treatment-specific spatially structured components were assigned intrinsic conditional autoregressive (CAR) prior distributions with precision hyperprior distributions of Gamma (0.5,0.005) [29, 30]. A sum-to-zero constraint was imposed on the random effects with intrinsic CAR prior distributions and the neighbours of SLA *i* were defined as the ones sharing a common geographical boundary.

The odds ratio is the odds of a particular factor compared with the odds of intended adjuvant therapies, with a larger odds ratio indicating the factor associated with higher odds of choosing particular adjuvant therapies. The shared effect odds ratio can be interpreted as the relative strength of the unobserved spatially related factors associated with a breast cancer patient’s choice of adjuvant therapy. These odds ratios can be presented as a thematic map to highlight the relationship between shared treatment effects and geographic location. A high shared effect odds ratio exp(*ϕ*_*i*_) indicates that women living in SLA *i* are more likely to access treatment facilities.

### Bayesian inference

Bayesian inference of the shared spatial component model was performed using Markov chain Monte Carlo (MCMC) algorithms as implemented in WinBUGS v1.4.3 [31] and interfaced with R v2.14.1 [32]. The full cohort Bayesian shared spatial component model was run with 2 chains for 17,500 iterations: 10,000 of the iterations were discarded as burn-in and a thinning factor of three was adopted, so that 5,000 iterations were retained for inference. The sub-cohort models were run with 37,500 iterations and a thinning factor of 3 to facilitate convergence for the smaller data samples, with the first 30,000 iterations discarded as burn-in.

Model convergence was examined using a range of diagnostics including the Gelman-Rubin statistics [33], trace plots, density plots and autocorrelation plots.

### Model evaluation

The Deviance Information Criterion (DIC) was used to determine how well a model fits the data, with smaller values indicating better model fit [34]. The DIC is calculated as the sum of the posterior mean of the deviance and an estimate of the effective number of parameters (pD), where smaller pD values indicate less complexity in the fitted model.

Posterior predictive checks (PPC) [34] were used to examine the adequacy of the model predictions compared with the observed data. These determine the percentage of the observed data within the 95 % credible interval of the corresponding posterior predictive distribution. For a given model, the posterior predictive distribution was formed by simulating data from the likelihood, for a given individual, based on a random selection of posterior samples. The estimated posterior odds ratio (here, posterior medians) was considered to be substantively different from the baseline if the 95 % credible interval (CrI) did not include unity [35–37].

Figures of SLA level QLD maps, with a set of common fixed cut-off values (<0.77, 0.91, 1.10, 1.30, 1.30 +) [38], were used to display the spatial odds ratio (exp(*ϕ*_*i*_)) shared by the three adjuvant therapies to assess the shared component effect. In addition, maps of the treatment-specific spatial effects (exp(*u*+*s*)) were also generated to assess the spatial variation in each adjuvant therapy. Only the sum of spatially structured *s* and unstructured random effect *u* is well-identified by the data [39], so it is common to map the sum of the spatially structured and unstructured random effects in shared component models (e.g. Earnest et al. [40]).

Maps of posterior probabilities that the spatial odds ratios exceeded unity were generated using the threshold rule proposed by Richardson et al. [41] to identify SLA with probability higher than 0.8 (respectively lower than 0.2), which can be considered as having an excess odds ratio (respectively a low odds ratio) with little uncertainty. The relative weight or influence of the common spatial effect between two therapies was measured as the ratio of two corresponding weights, such as *δ*_2_/*δ*_1_, *δ*_3_/*δ*_1_ and *δ*_3_/*δ*_2_, where *δ*_1_ is the weight for radiotherapy, *δ*_2_ is the weight for chemotherapy and *δ*_3_ is the weight for hormonal therapy.

The box plots are computed using the posterior samples for the respective parameters and reflect the general patterns in the estimated posterior median shared spatial effect across the geographic category of travel time to cancer care facilities and socio-economic status. These plots are compared with the Queensland average (i.e. above or below the vertical red line of QLD average = 1) within each geographic category, so should not be compared against one another. The box plots are in log-scale and the rectangular box within the box plot contains 50 % of the estimates. Two percentage columns for each geographic category were also included in the box plots. The left column represents the percentage of SLAs with less than 20 % posterior probabilities that the spatial odds ratios exceeded unity. The right column presents the percentage of SLAs with more than 80 % probabilities that the spatial odds ratios exceeded unity.

Finally, two other assessments of model robustness were made. First, the sensitivity of the model to the choice of priors was evaluated by assessing the change in the posterior parameter estimates to changes in the hyperparameters of selected prior distributions. Secondly, the model itself was challenged by proposing a range of alterations to the baseline model and evaluating resultant changes in the posterior parameter estimates with corresponding 95 % CrI and model DIC values.

### Model formulation

Seven alternative models were created based on the baseline Bayesian shared spatial component model (A0) as described by Eqs. 2 and ??. The full cohort data were used to examine each of these alternative models as listed below:

1. The spatially unstructured random effect *u* for each adjuvant therapy was assigned an independent Gaussian prior distribution.
2. A second shared component was added and shared between radiotherapy and chemotherapy.
3. A second shared component was added and shared between radiotherapy and hormonal therapy.
4. A second shared component was added and shared between chemotherapy and hormonal therapy.
5. The spatially unstructured random effect *u* was removed.
6. The spatially structured random effect *s* was removed.
7. The approximates non-informative or flat priors of zero mean Gaussian distribution with a constant low precision of 0.0001 was assigned to the intercept (*α*_*k*_) and regression coefficients (*β*_*k*_).

## Results

In this section, the results of fitting the baseline model A0 and the corresponding inferences are described in detail. The results of the other models A1 to A7 are then described and compared to the baseline results.

### Baseline model results

The Bayesian shared spatial component model (previously described) leads to different posterior estimations of the covariates effect depending on the intended treatment (Table 2). For all treatments, there was a suggested trend of decreased use with increasing travel time. This was particularly marked for radiotherapy when travel time to the closest radiation facility was above 4 h compared to less than one h (OR = 0.41, 95 % CrI: [0.23,0.74]). Intention to use radiotherapy or hormone therapy was similar across socio-economic status, while the suggestion of decreasing intention to use chemotherapy in more disadvantaged areas was compromised by imprecise estimates with wide credible intervals. As age increased there was clear evidence of decreased use of chemotherapy but increased use of hormonal therapy. There appeared to be less use of radiotherapy with increasing age, but only the oldest age group had substantively lower odds (Table 2). Women diagnosed with advanced stage breast cancer were more likely to intend to undertake radiotherapy and chemotherapy and less likely to choose hormonal therapy. For women with an unknown stage tumour, the intention to use chemotherapy was substantively higher than for localised tumour patients. Members of the study cohort diagnosed with breast cancer in their first attendance at mammography screening were generally substantively less likely to select any of the adjuvant therapies than women who had attended previous mammograms before their cancer diagnosis. The intended choice of surgical procedure had an obvious influence on the choice of adjuvant therapy, where patients choosing to have mastectomy were substantively less likely to have radiotherapy or hormonal therapy before or after the surgery, but were substantively more likely to have chemotherapy.

**Table 2:** Estimated posterior odds ratios of patient characteristics associated with the intended adjuvant therapies, and relative weights between therapies

		Median posterior odds ratios [95 % CrI^a^]		
Factors	N	Radiotherapy	Chemotherapy	Hormonal Therapy
Road travelling time (TRACT)				
<1 h	4514	1.00	1.00	1.00
1−<2 h	502	0.88 [0.56, 1.39]	0.74 [0.46, 1.19]	0.94 [0.68, 1.31]
2−<4 h	750	0.66 [0.42, 1.04]	0.91 [0.58, 1.45]	0.97 [0.70, 1.35]
4−<6 h	317	0.41 [0.23, 0.74]	0.44 [0.25, 0.81]	0.74 [0.49, 1.08]
6 or more h	274	0.41 [0.24, 0.73]	0.60 [0.34, 1.07]	0.71 [0.48, 1.04]
Socio-economic status (IRSAD)				
Quintile 1 Most disadvantaged	817	0.94 [0.65, 1.36]	0.73 [0.50, 1.04]	1.01 [0.77, 1.32]
Quintile 2	1458	0.80 [0.56, 1.12]	0.86 [0.62, 1.19]	0.91 [0.71, 1.15]
Quintile 3	1762	1.03 [0.76, 1.39]	0.91 [0.69, 1.20]	1.01 [0.81, 1.26]
Quintile 4	1539	0.92 [0.68, 1.24]	0.93 [0.70, 1.22]	1.03 [0.83, 1.28]
Quintile 5 Most advantaged	781	1.00	1.00	1.00
Age at diagnosis (years)				
40–49	933	1.07 [0.85, 1.34]	1.85 [1.51, 2.27]	0.83 [0.71, 0.98]
50–59	2064	1.00	1.00	1.00
60–69	2074	0.86 [0.72, 1.03]	0.50 [0.42, 0.60]	1.20 [1.06, 1.37]
70–89	1225	0.36 [0.29, 0.44]	0.12 [0.09, 0.16]	1.39 [1.18, 1.63]
Indigenous status				
Non-Indigenous	5467	1.00	1.00	1.00
Indigenous	63	1.66 [0.83, 3.49]	1.16 [0.61, 2.17]	1.33 [0.89, 2.04]
Indigenous unknown	766	0.87 [0.69, 1.10]	0.40 [0.30, 0.53]	1.06 [0.89, 1.26]
Marital status				
Married	4150	1.00	1.00	1.00
Never married	305	0.78 [0.57, 1.09]	0.79 [0.56, 1.10]	0.85 [0.68, 1.08]
Widowed/Divorced/Separated	1665	0.83 [0.71, 0.99]	0.95 [0.80, 1.14]	1.05 [0.92, 1.19]
Marital unknown	176	0.91 [0.59, 1.44]	1.05 [0.62, 1.73]	0.91 [0.67, 1.24]
Tumour stage				
Localised (Stage I)	4081	1.00	1.00	1.00
Advanced (Stage II, III, IV)	2139	2.43 [2.03, 2.90]	11.21 [9.60, 13.05]	0.87 [0.77, 0.97]
Stage unknown	76	0.59 [0.32, 1.08]	2.83 [1.42, 5.42]	1.24 [0.84, 1.88]
Occupation				
Blue collar	245	1.71 [1.18, 2.48]	1.03 [0.71, 1.48]	0.98 [0.76, 1.27]
White collar	907	1.48 [1.18, 1.87]	1.19 [0.96, 1.47]	1.11 [0.95, 1.31]
Professional	1061	1.49 [1.21, 1.84]	1.38 [1.13, 1.71]	1.09 [0.94, 1.28]
Not in the labour force	2631	1.00	1.00	1.00
Unknown	1452	1.28 [1.06, 1.55]	1.05 [0.85, 1.29]	1.27 [1.10, 1.47]
Invasive tumour type				
Invasive ductal	4074	1.00	1.00	1.00
Tubular	167	0.48 [0.33, 0.71]	0.27 [0.12, 0.55]	0.91 [0.68, 1.21]
Lobular classical	506	0.95 [0.74, 1.22]	0.73 [0.56, 0.95]	1.72 [1.40, 2.12]
Other	434	0.73 [0.56, 0.96]	0.77 [0.57, 1.03]	0.94 [0.78, 1.15]
Unknown	1115	0.81 [0.67, 0.98]	0.69 [0.57, 0.84]	0.70 [0.61, 0.80]

Table 2 also shows the estimated posterior median value (and its uncertainty) of the relative influence, or level of importance, of the shared component effect between adjuvant therapies. The shared effect had the greatest relative influence (highest level of importance) to both radiotherapy and hormonal therapy, and had least influence (lowest level of importance) on chemotherapy.

**Fig. 2:**
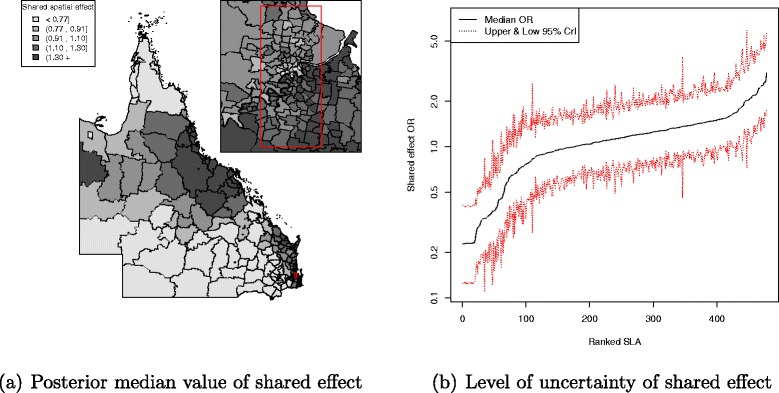
Posterior odds ratio for the shared component effect among adjuvant therapies at SLA level, 1997–2008. Estimated posterior odds ratio for the 478 SLA shared spatial effect to the intended adjuvant therapies among all study cohort across QLD. Map (**a**) with median value of the shared spatial effect separated into quintiles (<0.77, 0.91, 1.10, 1.30, 1.30 +) and a line plot (**b**) for the ranked SLA median shared spatial effect values with 95 % credible interval

**Fig. 3:**
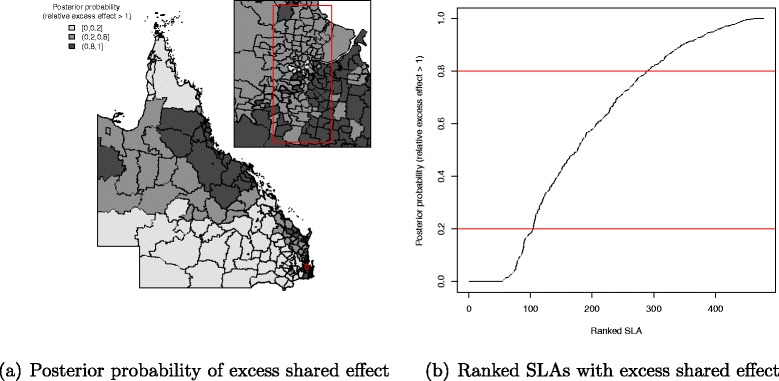
Posterior probability of excess shared component effect among adjuvant therapies at SLA level, 1997–2008. Map (**a**) posterior probability of excess shared component effect (less than 0.2, 0.2–0.8 and greater than 0.8) and a line plot (**b**) approximate number of ranked SLAs with excess shared component effect in QLD

**Fig. 4:**
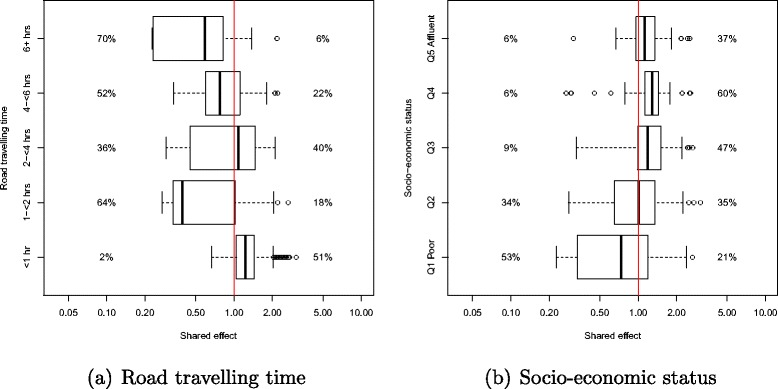
Box plots of the median shared spatial effect and percentage of SLA with less than 20 % (to the left of box plot) or more than 80 % (to the right of box plot) probability that shared effect odds ratios exceeded unity by (**a**) road travelling time and (**b**) socio-economic status

**Fig. 5:**
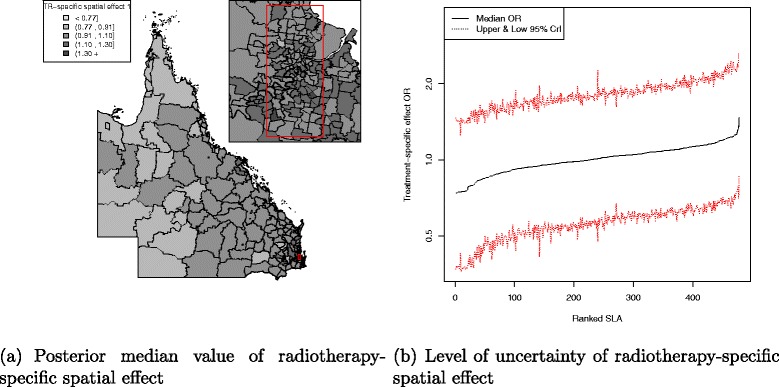
Posterior odds ratio for the radiotherapy-specific spatial effect at SLA level, 1997–2008. Estimated posterior odds ratio for the 478 SLA radiotherapy-specific spatial effect among all study cohort across QLD. Map (**a**) with median value of the radiotherapy-specific spatial effect separated into quintiles (<0.77, 0.91, 1.10, 1.30, 1.30 +) and a line plot (**b**) for the ranked SLA median radiotherapy-specific spatial effect values with 95 % credible interval

**Fig. 6:**
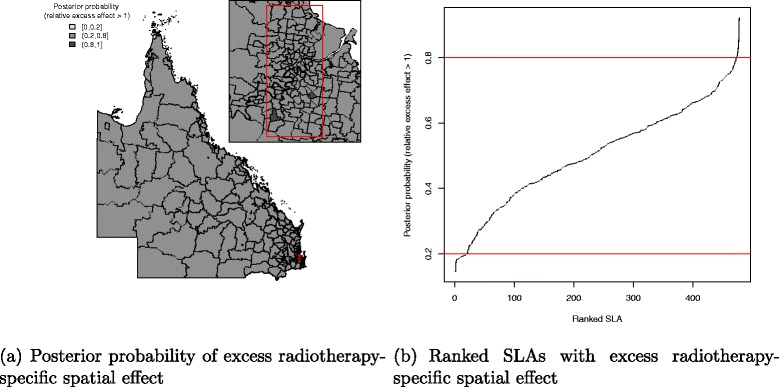
Posterior probability of excess radiotherapy-specific spatial effect at SLA level, 1997–2008. Map (**a**) posterior probability of excess radiotherapy-specific spatial effect (less than 0.2, 0.2–0.8 and greater than 0.8) and a line plot (**b**) approximate number of ranked SLAs with excess radiotherapy-specific spatial effect in QLD

**Fig. 7:**
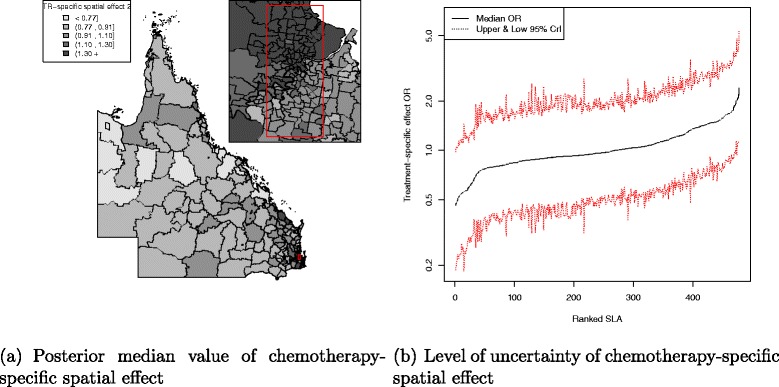
Posterior odds ratio for the hormonal-specific spatial effect at SLA level, 1997–2008. Estimated posterior odds ratio for the 478 SLA hormonal-specific spatial effect among all study cohort across QLD. Map (**a**) with median value of the hormonal-specific spatial effect separated into quintiles (<0.77, 0.91, 1.10, 1.30, 1.30 +) and a line plot (**b**) for the ranked SLA median hormonal-specific spatial effect values with 95 % credible interval

**Fig. 8:**
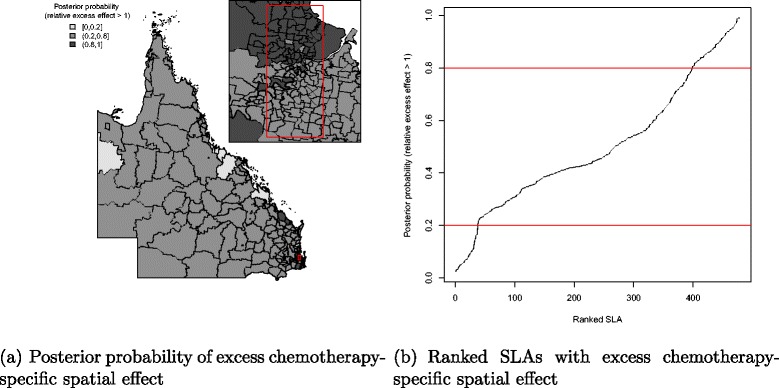
Posterior probability of excess hormonal-specific spatial effect at SLA level, 1997–2008. Map (**a**) posterior probability of excess hormonal-specific spatial effect (less than 0.2, 0.2–0.8 and greater than 0.8) and a line plot (**b**) approximate number of ranked SLAs with excess hormonal-specific spatial effect in QLD

**Fig. 9:**
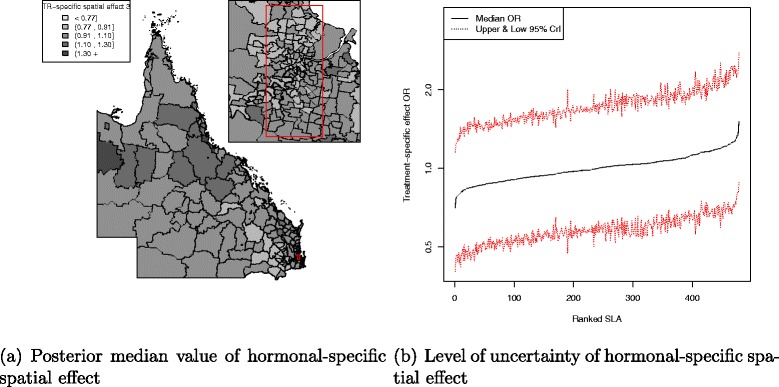
Posterior odds ratio for the chemotherapy-specific spatial effect at SLA level, 1997–2008. Estimated posterior odds ratio for the 478 SLA chemotherapy-specific spatial effect among all study cohort across QLD. Map (**a**) with median value of the chemotherapy-specific spatial effect separated into quintiles (<0.77, 0.91, 1.10, 1.30, 1.30 +) and a line plot (**b**) for the ranked SLA median chemotherapy-specific spatial effect values with 95 % credible interval

**Fig. 10:**
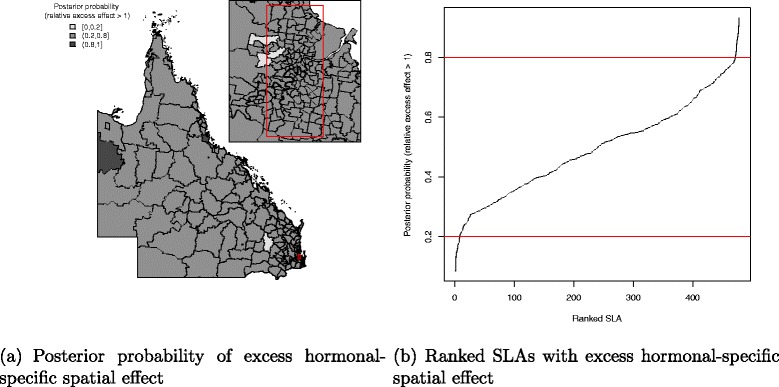
Posterior probability of excess chemotherapy-specific spatial effect at SLA level, 1997–2008. Map (**a**) posterior probability of excess chemotherapy-specific spatial effect (less than 0.2, 0.2–0.8 and greater than 0.8) and a line plot (**b**) approximate number of ranked SLAs with excess chemotherapy-specific spatial effect in QLD

There was a pattern of increasing shared component effect toward regions of concentrated radiation facilities in the north-east (Townsville) and south-east (Brisbane) of Queensland (Fig. 2a). Plots of the posterior probability of excess shared effect are shown in Fig. 3. From Fig. 4, there is an indication of geographic differences in the posterior shared component effect. Not only do many of the SLAs within less than 1 h travel time have larger median values, but many of these have probabilities above 80 % of genuinely being above the Queensland average. Likewise, the lower median values for many of the SLAs in areas with more than six h of travel time was also supported by the posterior probabilities suggesting the majority have values below the Queensland average. Similarly, for the socio-economic status there is an indication of more affluent regions having higher values of the shared component effect than the Queensland average. This was also demonstrated by the SLA percentage columns as more affluent areas had much higher percentage of SLA in the right hand column than the left hand column. There was no obvious radiotherapy-specific and hormonal therapy spatial effect pattern across Queensland as shown in both Figs. 5 and 6 and both Figs. 7 and 8 respectively. The chemotherapy-specific spatial effect plots (Figs. 9 and 10) shows that regions with higher spatial variation appear to be around south-east of Queensland. Since the 95 % credible intervals include zero under the baseline model A0, it can be argued that there is no substantive correlation between adjuvant therapies described by the treatment-specific unstructured random effects (see Additional file 1: Table S1).

**Table 2:** Estimated posterior odds ratios of patient characteristics associated with the intended adjuvant therapies, and relative weights between therapies *(Continued)*

First screen diagnosed				
Yes	1508	0.81 [0.69, 0.96]	0.79 [0.66, 0.94]	0.72 [0.63, 0.82]
No	4788	1.00	1.00	1.00
Surgery				
Breast-conserving surgery	4255	1.00	1.00	1.00
Mastectomy	2009	0.03 [0.02, 0.03]	1.63 [1.40, 1.91]	0.86 [0.76, 0.98]
No surgery	15	0.15 [0.04, 0.44]	2.26 [0.76, 6.61]	0.86 [0.49, 1.44]
Unknown	17	0.18 [0.06, 0.47]	1.70 [0.64, 4.71]	1.10 [0.66, 1.89]
Relative weight of shared component				
Radiotherapy		1.00	—	—
Chemotherapy		0.57 [0.36, 0.84]	1.00	—
Hormonal therapy		0.90 [0.68, 1.17]	1.59 [1.09, 2.47]	1.00
PPC	0.9993			

^a^ *Abbreviations*: *CrI* Credible interval, *N* Number of patients, *TRACT* Travel to cancer treatment, *IRSAD* Index of relative socio-economic advantage and disadvantage, *PPC* Posterior predictive check

The analysis was repeated by cohort in each broad age group at diagnosis and tumour stage categories to further examine the variation in the shared component. The analysis of each separate cohort was not adjusted for the corresponding predictor variable; for instance, the model analysis of the 40−49 age group cohort does not adjust for age at diagnosis variable but is adjusted for all other variables specified in the Data description section above. When the study cohort was analysed by age group and tumour stage at diagnosis (Table 3), radiotherapy and hormonal therapy remained the most influential treatments on the shared component in the 50−59 years age group and chemotherapy was still the least influential. In the 70−89 years age group, all adjuvant therapies were equally influential on the shared component. The analysis by cohort of localised tumour stage at diagnosis showed a relative weight pattern similar to that found for the 50−59 year old cohort, whereas the shared component was equally influenced by any of the studied adjuvant therapies in advanced tumour patients.

**Table 3:** Relative weight of shared component between adjuvant therapies for separate cohort model adjusted for all other predictor variables

	Median posterior relative weights [95 % CrI^a^]		
	Radiotherapy	Chemotherapy	Hormonal therapy
At age 40–49			
Radiotherapy	1.00	—	—
Chemotherapy	0.57 [0.31, 0.94]	1.00	—
Hormonal therapy	0.86 [0.57, 1.29]	1.51 [0.92, 2.66]	1.00
At age 50–59			
Radiotherapy	1.00	—	—
Chemotherapy	0.40 [0.23, 0.65]	1.00	—
Hormonal therapy	0.87 [0.63, 1.19]	2.16 [1.36, 3.63]	1.00
At age 60–69			
Radiotherapy	1.00	—	—
Chemotherapy	0.58 [0.31, 0.95]	1.00	—
Hormonal therapy	0.81 [0.58, 1.11]	1.39 [0.84, 2.62]	1.00
At age 70–89			
Radiotherapy	1.00	—	—
Chemotherapy	0.92 [0.48, 1.55]	1.00	—
Hormonal therapy	0.74 [0.50, 1.08]	0.80 [0.48, 1.55]	1.00
Localised tumour			
Radiotherapy	1.00	—	—
Chemotherapy	0.34 [0.18, 0.58]	1.00	—
Hormonal therapy	0.81 [0.61, 1.05]	2.42 [1.39, 4.47]	1.00
Advanced tumour			
Radiotherapy	1.00	—	—
Chemotherapy	0.86 [0.63, 1.18]	1.00	—
Hormonal therapy	0.86 [0.61, 1.17]	0.99 [0.72, 1.41]	1.00

^a^Credible interval

The trends for the shared component effect in each age group and tumour stage (see Additional file 2: Figure S1–S6) were all similar to the map shown in Fig. 2 which has an increasing effect towards the north-east (Townsville) and south-east (Brisbane) regions of Queensland. In general, both road travelling time and socio-economic status box plots for the posterior shared component effect proportion in each sub-cohort model (age group and tumour stage categories) have patterns similar to the plots in Fig. 4 (see Additional file 2: Figure S7–S8).

With respect to model checking, the model converged with Gelman-Rubin statistics very close to unity for all parameters. Acceptable convergence was further supported by the trace and autocorrelation plots. In addition, the posterior predictive checks (PPC) for the baseline Bayesian model also gave values very close to unity (Table 2), which confirmed that the model fitted the data adequately.

Sensitivity analysis was performed to evaluate the choice of the priors in the model. The precision parameters of *α*, *β*, *s* and *ϕ* were given Gamma (0.5,0.5) and Gamma (0.5,0.05) hyperprior distributions, and a less informative normal prior for log (*δ*), with a precision of 2.9, was also considered. The results were insensitive to the prior choice for all fixed effects, shared spatial effects, and treatment-specific effects for all adjuvant therapies, except for the hormonal therapy whose treatment-specific spatial effect was slightly sensitive to the choice of precision. This indicates that there is sufficient information in the data sources to learn about the fixed effects *β*, shared spatial component *ϕ* and the treatment-specific spatial components *s*. The weight *δ* for all the adjuvant therapies is insensitive to the less informative *δ* prior (see Additional file 2: Figure S9). However, the weight for hormonal therapy showed some sensitivity to the hyperprior choice of the treatment-specific spatial effects *s*. The less informative precision distribution of Gamma (0.5,0.5) for *s* caused the 95 % CrI of the relative weight ratio between hormonal therapy and chemotherapy (*δ*_3_/*δ*_2_) to change from substantive to not substantive. The more informative precision prior will force the *s* to be close to zero, which suggests that by excluding *s*, the hormonal therapy treatment-specific spatial variation was then forced into the shared component.

### Other model results

All the alternative models (A1–A7) results were assessed in the same way as the baseline model A0 to facilitate comparison (see Additional file 1: Table S3–S9). Posterior estimates of the covariate effects for each intended treatment were mostly similar to the baseline model A0. All of these alternative models (A1–A7) had similar substantial posterior estimates for part of the variable of interest compared to the baseline model A0, such as socio-economic status, age at diagnosis, Indigenous status, marital status, tumour stage, occupation, invasive tumour type, first screen diagnosed and the intended surgical procedure. Here we describe the key differences between the alternative model results compared to the baseline model A0.

Compared with the baseline model A0, fitting model A1 (assigning independent Gaussian distribution to the unstructured random effects ‘*u*’) resulted in no substantial change to the posterior estimate of the fixed effects, the relative weight between treatments, or the shared and treatment-specific spatial components (see Additional file 1: Table S3). The key difference was that this model had poorer fit to the data with a substantively larger DIC value compared to the baseline model A0 (see Additional file 1: Table S2).

Of the three alternative models A2, A3 and A4 with a second shared component allocated to two out of three adjuvant therapies, all had similar posterior estimates to the baseline model A0, with the second shared component explaining only a very little amount of spatial variation (see Additional file 1: Table S4–S6). The A3 model, with the second shared component allocated to radiotherapy and hormonal therapy, had the key difference that all the relative weights for the first shared component had a 95 % CrI that included unity, which is substantially different to the baseline model A0. The fit of models A2 and A4 was poorer than the baseline model A0 with higher DIC values, while model A3 had a similar DIC value to the baseline model A0 (see Additional file 1: Table S2).

As for the model (A5) without a spatially unstructured random effects term ‘*u*’, the comparison with the baseline model A0 showed similar posterior estimates of the regression coefficients of interest (see Additional file 1: Table S7). However, the excess treatment-specific spatially structured error term ‘*s*’ increased, which in turn inflated the proportion of SLAs with excess odds ratios (computed as exp(*s*) under this model). The increase was most obvious for the hormonal therapy specific spatially structured random effect which had the proportion of SLAs with excess odds ratios increase from 3 % in the baseline model to 28 % in model A5. Model A5 had a substantially lower DIC value of 18,254 than the baseline model (DIC = 18,364), indicating better model fit (see Additional file 1: Table S2).

Under model A6, without a spatially structured random effects term ‘*s*’, the posterior estimates changed substantially within the TRACT variable for the radiotherapy and chemotherapy. Patients in the TRACT categories of more than 2 h were substantially less likely to use radiotherapy compared with those who only needed to travel within an hour to a treatment facility (see Additional file 1: Table S8). The intention to use chemotherapy was in general substantially less in all TRACT categories compared to those with less than an hour of travelling time, except the 2−<4 h category had similar odds ratio to the reference category. One additional difference to the baseline model A0 is that the most disadvantaged socio-economic (Quintile 1) patients were substantially less likely to intended to use chemotherapy than the most advantaged (Quintile 5) patients. This alternative model also had the shared component capture more spatial variation in the absence of ‘*s*’ as compared to the baseline model, and there was virtually no excess treatment-specific spatial variability captured by the unstructured random effect ‘*u*’. Model A6 did not fit the data better than the baseline model with a substantially higher DIC value (see Additional file 1: Table S2).

The use of approximate non-informative or flat priors with relatively small constant values for the precision (Normal (0,0.0001)) in model A7 gave substantively different results in the posterior estimate for the TRACT variable across all adjuvant therapies as compared to the baseline model. In this alternative model, all three therapies presented a decreasing trend of posterior odds ratios with increasing road travelling time (TRACT), with radiotherapy and hormonal therapy had substantially lower odds ratios for those who had more than 2 h of travelling time, and chemotherapy had substantively lower odds ratios for patients with more than 4 h of travelling time (see Additional file 1: Table S9). In addition, the 95 % CrI of relative weight between chemotherapy and hormonal therapy had changed from substantial in the baseline model to not substantial in model A7. This flat prior model (A7) had a DIC value of 18,367 with pD = 422 that was not substantially different to the baseline model (DIC = 18,364, pD = 417) (see Additional file 1: Table S2).

## Discussion and conclusions

To the best of our knowledge, this is the first study to examine the shared spatial disparities of women’s intention to use adjuvant therapy after being diagnosed with screen-detected breast cancer using Bayesian spatial modelling techniques. In this study we utilised a shared spatial-components modelling strategy to quantify what factors influence a woman’s intended adjuvant treatment for screen-detected breast cancer in Queensland. The analysed results were concluded based on the baseline model A0, unless otherwise specified. We found that the intention to use adjuvant therapy varied by geographical location, with women living in regions having closer access to a cancer treatment facility being more likely to intend to use adjuvant therapy than those who lived further away. A clear increased posterior median shared spatial effect was observed towards the north-east (Townsville) and south-east (Brisbane) regions of Queensland with little uncertainty, supported by the posterior probability map.

Previous studies have shown that the breast cancer treatment that a woman choose to undertake is influenced by ease of access to treatment [7, 14, 15, 19]. Our analysis, based on the intended treatment, found similar geographic differentials. This suggests that women may be making decisions regarding their treatment strategy based on perceived barriers to treatment, not simply an inability to take up their intended option. It is not clear whether this reflects the decision making of women themselves, or the recommendations made by their referring doctor, and the lack of actual treatment information makes more substantive interpretation impossible.

The use of a shared spatial component is one of the appealing features of the model as it permits isolation of clusters of areas of common variation for the three adjuvant therapies. This latent variable which is shared among all adjuvant therapies is spatially structured, following a specific intrinsic conditional autoregressive (CAR) model. This means that the shared component acts as a surrogate for some unobserved spatially structured factors that may explain the geographical variations of the usage of the three adjuvant therapies (radiotherapy, chemotherapy, hormonal therapy) of interest. Given the strong association with distance to tertiary hospitals, it appears that the shared term reflects the ease of access to treatment facilities. This could also reflect the several barriers or considerations associated with undergoing treatment, such as waiting time to treatments, burden of travel, being away from home, lack of closeness to family and friends, work and family demands, financial burden, feeling of being a burden on others, cost-effectiveness of treatments and patient’s medication-taking behaviour, as indicated by previous studies [8, 12, 42, 43]. Thus the shared spatial effect maps are consistent with an increasing ease of access to adjuvant treatment facilities towards the north-east (Townsville) and south-east (Brisbane) regions of Queensland. In addition to the shared component, there remain some potential sources of treatment-specific effect for chemotherapy in the data. However, at present, it is unclear what is causing this finding.

While the shared effect has a similar trend for the entire study cohort as well as the age and tumour stage at diagnosis subgroups, the patterns of relative weight, or level of importance, of the shared component between adjuvant therapies were not all the same. The ease of access to treatment facilities barrier seems to be more influential in the sub-cohort of age 50−59 years or with localised tumour stage than any other subgroups. No such differences in relative weight among adjuvant therapies were detected for the higher risk cohorts of age 70−89 or with advanced stage tumour. This could suggest that the higher risk cohorts, with more urgent need to have treatment, would be more likely to overcome common barriers between adjuvant therapies. Meanwhile, the absence of evidence of a difference in relative weight among adjuvant therapies could also be due to a lack of signal in the data since only sub-cohorts of patients are considered. The recommendation for hormone therapy is not determined by tumour type, stage or surgery but by ER/PR status, which is independent of age [44, 45]. As such, our finding of a fairly similar influence of the shared spatial effect between hormonal therapy and radiotherapy was surprising. While it could be due to the effect of administering hormonal therapy via surgical treatment, rather than ongoing self-administered oral medication, the ability to detect differences in relative weight among adjuvant therapies may be compromised due to a lack of more specific information about the intended treatment.

The relationship between patient characteristics and the intended choice of adjuvant therapies is consistent with most other international studies. The intended choice of adjuvant therapies was not associated with socio-economic status; this is similar to the British Columbia study [46] which found no significant differences in the use of chemotherapy or hormonal therapy by population size of local health authorities, while the use of chemotherapy in the United States of America seems to be influenced by socio-economic factors like poverty among patients ages 65−69 years [18]. A more recent study by Ursem et al. [47] also suggested that low income patients may have low use of adjuvant endocrine therapy. The finding of reduced likelihood of choosing radiotherapy with increased travel time in this study supports other studies in the United States of America [15, 16], which showed that rural area patients were less likely to receive radiotherapy following lumpectomy or mastectomy than their urban counterparts. Both radiotherapy and chemotherapy had a decreased likelihood of being chosen with increased age at diagnosis, but this relationship was reversed for hormonal therapy. This relationship between a patient’s age and the use of radiotherapy in Queensland is again consistent with studies in the United States of America [15, 16] that found increasing age was associated with decreased likelihood of receiving post-mastectomy radiation or post-lumpectomy radiation. Radiotherapy and chemotherapy were also more likely to be the choice for advanced stage tumour patients, but this cohort was less likely to consider hormonal therapy. Some published evidence [13, 46, 48] has shown that women living in remote areas tended to favour mastectomy, which may impact on the selection of adjuvant therapies to prevent the need to travel to a cancer treatment center that is far away. This phenomenon is particularly strong for the selection of radiotherapy and marginally influential for hormonal therapy, but the selection of chemotherapy has an opposite relationship.

The alternative models (A1–A7) provided some different results as compared to the baseline model A0. By using an independent Gaussian distribution on the unstructured random effect ‘*u*’, the results from model A1 were not substantially different to those of the baseline model, despite a poorer fit to the data (larger DIC value). This suggests the use of a simple structure prior distribution only on the unstructured random effects reduces the model’s performance in fitting the data. For the three alternative models A2, A3 and A4, the second shared component explained only a very small amount of the spatial variability. This spatial variability was likely to be explained as part of the treatment-specific components in the baseline model. This implies that the model does not require an additional shared component.

A model without a spatially unstructured random effects term ‘*u*’ (A5) and a model without a spatially structured random effects term ‘*s*’ (A6) was examined. Compared with the baseline model which included both of these terms (A0), we found that model A5 gave similar posterior estimates of the effects (regression coefficients) of interest, but the spatially structured error term increased (presumably to accommodate the lack of the unstructured error term), which in turn inflated the proportion of SLAs with excess odds ratios (computed as exp(*s*) under this model). This inflation is interesting, since it implies that the single (spatial) error term is larger than the two error terms in the baseline model. Hence this model appears to draw out some of the partially spatial information that is incorporated in the covariates under the baseline model, in order to provide an equivalent fit to the data. Since we do not feel that this behaviour is optimal, and that including the spatially unstructured random effect term had better theoretical grounds than excluding it, we preferred to focus on the results of this A0 model rather than the more speculative model A5. Under model A6, the excess spatial information that was attributed to the spatial random effects term in the baseline model appeared to be attributed instead to the stronger spatial covariates, in particular TRACT and the corresponding shared component. Interestingly, under this model the TRACT regression parameters indicated that all of the treatment choices were affected by distance from the treatment centre. This differs from the results of the baseline model, which were that TRACT was a substantive factor only in the choice of radiotherapy. Thus it seems here that the lack of a specific spatial term in the model induces greater spatial variation in the parameters that have a strong spatial signal. This has interesting implications for spatial modelling in general to support the inclusion of spatial random effect, and this case study in particular. We have also investigated a seventh alternative model (A7), which approximates non-informative or flat priors by imposing a relatively small constant value for the precisions for these prior distributions. This resulted in substantially different results as compared to the baseline model, where model A7 had a decreased odds ratios trend in the TRACT variable with increasing travelling for all the intended adjuvant therapies. The universal decreasing trend of treatment choice with increasing travel time to closest radiation facility for all adjuvant therapies may be unrealistic at least for hormonal therapy. However, given the limited data in the QLD context, this is difficult to justify.

This study had several limitations resulting from using routinely collected data. One limitation was the lack of data on actual treatment received and/or completed. The use of intended treatment data may not reflect the actual treatment undertaken, or it may not have been completed. Another limitation is that the treatment data were restricted to screen-detected breast cancer patients, rather than all breast cancer patients. Although the treatment recommendations by Cancer Australia [45] are not specific to the detection method, it is not known whether women who access public mammography screening would experience the same perceived barriers to treatment as women who access either no screening or access screening through private providers. Finally, the proposed Bayesian shared spatial component models have a complex structure with the inclusion of CAR latent structures and multivariate priors. This complexity has the drawback of increased computational demands, in particular longer simulation time and careful assessment of MCMC convergence. It is noted that the PPC is an evaluative diagnostic that, while informative as an indicator of model fit, may tend to lead to optimistic conclusions since the data used to validate the model are the same as those used to fit the model.

The primary purpose of most population-based cancer registries is to collect information about the number and characteristics of incident cancers. As such, the level of detail available in these registries about subsequent management is often limited. Some studies [9, 13, 49] have used data linkage to hospital admitted patient data collections to access information about surgical treatment. However, these data collections typically do not include information about adjuvant treatment which can be administered in hospital outpatient and other types of clinical practices. Therefore it is difficult to gain an understanding of the geographic variation in actual treatment uptake and completion. For this reason, while there are limitations in using intended, rather than actual treatment as the outcome variable, these data advance our understanding of geographical variations in intention.

The purpose of this study is to identify and quantify how the shared component effect influences intended choice between all three adjuvant therapies. It is not to assess how the intended choice of each individual adjuvant therapy was influenced by patient’s characteristics. Hence no separate model was fitted for each adjuvant therapy and compared to the Bayesian shared spatial model.

In conclusion, this study has identified several important results. The choice of adjuvant therapy, particularly radiotherapy, was generally strongly associated with the distance to radiotherapy treatment facilities. Older patients have substantively lower intention to use radiotherapy and chemotherapy, instead preferring hormonal therapy. In contrast, those with advanced cancers tend to choose radiotherapy and chemotherapy, even though the current treatment recommendations for advanced breast cancer [45] also recommend hormonal therapy. Moreover, even after adjusting for key demographic and clinical factors, the presence of residual shared spatial effects indicates that there are other unmeasured geographical barriers influencing women’s treatment choices. This highlights the need to identify the additional barriers that impact on treatment intentions among women diagnosed with screen-detected breast cancer, particularly for those women living further away from cancer treatment centers.

## Additional files

Additional file 1
**Supplementary Tables.** (PDF 88 kb)

Additional file 2
**Supplementary Figures.** (ZIP 28672 kb)
