# Supplementary material for: Geographic variation in the intended choice of adjuvant treatments for women diagnosed with screen-detected breast cancer in Queensland
Source: BMC Public Health. 2015 Dec 2;15:1204. doi: 10.1186/s12889-015-2527-2 (PMC4668608; doi:10.1186/s12889-015-2527-2)
Supplement: Additional file 1 — Supplementary Tables. (PDF 88 kb) [file 12889_2015_2527_MOESM1_ESM.pdf]

Table 1: Estimated posterior correlation of the treatment-specific unstructured random effects between adjuvant therapies.

**Median posterior correlation [95% CrI[a]]**	**Radiotherapy**	**Chemotherapy**	**Hormonal Therapy**
Radiotherapy	15.82 [9.09, 25.51]	—	—
Chemotherapy	-0.78 [-6.62, 4.88]	13.49 [7.92, 22.86]	—
Hormonal therapy	-2.41 [-9.10, 3.62]	0.69 [-5.03, 6.54]	18.51 [11.95, 29.81]

[a]Abbreviations: CrI=Credible interval.

Table 2: Model Comparison.

**Model**	**pD[a]**	**DIC[b]**
(baseline) A0	417	18364
(spatially unstructured random effect 'u' with independent Gaussian prior) A1	434	18380
(second shared component for radiotherapy and chemotherapy) A2	422	18370
(second shared component for radiotherapy and hormonal therapy) A3	413	18363
(second shared component for chemotherapy and hormonal therapy) A4	422	18371
(without spatially unstructured random effect 'u') A5	294	18254
(without spatially unstructured random effect 's') A6	397	18380
(flat prior for intercept and regression coefficients) A7	425	18369

[a]Abbreviations: pD=Effective number of parameters.
[b]Abbreviations: DIC=Deviance Information Criterion.

Table 3: Model A1 (independent Gaussian prior for 'u') for estimated posterior odds ratios of patient characteristics associated with the intended adjuvant therapies, and relative weights between therapies.

Factors	N	Median posterior odds ratios [95% CrI[a]]		
		**Radiotherapy**	**Chemotherapy**	**Hormonal Therapy**
**Road travelling time (TRACT)**				
< 1 hour	4514	1.00	1.00	1.00
1– < 2 hours	502	0.88 [0.55, 1.39]	0.75 [0.48, 1.19]	0.93 [0.67, 1.31]
2– < 4 hours	750	0.65 [0.42, 1.04]	0.92 [0.60, 1.44]	0.99 [0.71, 1.36]
4– < 6 hours	317	0.41 [0.24, 0.73]	0.44 [0.24, 0.80]	0.74 [0.49, 1.08]
6 or more hours	274	0.41 [0.23, 0.70]	0.60 [0.34, 1.07]	0.72 [0.49, 1.04]
**Socio-economic status (IRSAD)**				
Quintile 1 Most disadvantaged	817	0.93 [0.64, 1.36]	0.72 [0.50, 1.04]	1.02 [0.78, 1.34]
Quintile 2	1458	0.80 [0.57, 1.10]	0.84 [0.60, 1.17]	0.91 [0.73, 1.16]
Quintile 3	1762	1.03 [0.76, 1.37]	0.90 [0.67, 1.19]	1.02 [0.82, 1.25]
Quintile 4	1539	0.91 [0.68, 1.22]	0.92 [0.68, 1.22]	1.04 [0.84, 1.28]
Quintile 5 Most advantaged	781	1.00	1.00	1.00
**Age at diagnosis (years)**				
40-49	933	1.07 [0.85, 1.34]	1.85 [1.52, 2.25]	0.83 [0.71, 0.98]
50-59	2064	1.00	1.00	1.00
60-69	2074	0.86 [0.72, 1.03]	0.50 [0.42, 0.60]	1.20 [1.05, 1.38]
70-89	1225	0.36 [0.29, 0.45]	0.12 [0.09, 0.16]	1.39 [1.19, 1.62]
**Indigenous Status**				
Non-Indigenous	5467	1.00	1.00	1.00
Indigenous	63	1.67 [0.83, 3.49]	1.16 [0.60, 2.19]	1.33 [0.88, 2.05]
Indigenous Unknown	766	0.87 [0.69, 1.09]	0.40 [0.30, 0.53]	1.06 [0.89, 1.26]
**Marital Status**				
Married	4150	1.00	1.00	1.00
Never Married	305	0.78 [0.56, 1.08]	0.80 [0.57, 1.11]	0.85 [0.67, 1.08]
Widowed/Divorced/Separated	1665	0.83 [0.71, 0.99]	0.95 [0.80, 1.14]	1.05 [0.93, 1.19]
Marital Unknown	176	0.92 [0.59, 1.44]	1.07 [0.63, 1.78]	0.91 [0.67, 1.24]
**Tumour Stage**				
Localised (Stage I)	4081	1.00	1.00	1.00
Advanced (Stage II, III, IV)	2139	2.43 [2.05, 2.91]	11.25 [9.67, 13.15]	0.87 [0.77, 0.98]
Stage Unknown	76	0.58 [0.31, 1.09]	2.83 [1.43, 5.42]	1.24 [0.84, 1.90]
**Occupation**				
Blue collar	245	1.71 [1.18, 2.50]	1.03 [0.71, 1.48]	0.98 [0.76, 1.26]
White collar	907	1.49 [1.19, 1.87]	1.19 [0.96, 1.47]	1.11 [0.94, 1.32]
Professional	1061	1.49 [1.22, 1.85]	1.38 [1.13, 1.71]	1.09 [0.94, 1.28]
Not in the labour force	2631	1.00	1.00	1.00
Unknown	1452	1.28 [1.06, 1.55]	1.04 [0.85, 1.28]	1.27 [1.10, 1.47]
**Invasive tumour type**				
Invasive Ductal	4074	1.00	1.00	1.00
Tubular	167	0.48 [0.33, 0.71]	0.27 [0.12, 0.55]	0.91 [0.68, 1.21]
Lobular Classical	506	0.95 [0.75, 1.22]	0.73 [0.55, 0.95]	1.72 [1.40, 2.13]
Other	434	0.73 [0.56, 0.98]	0.77 [0.56, 1.04]	0.95 [0.77, 1.15]
Unknown	1115	0.81 [0.67, 0.98]	0.69 [0.57, 0.84]	0.70 [0.61, 0.81]
**First screen diagnosed**				
Yes	1508	0.81 [0.68, 0.96]	0.78 [0.66, 0.93]	0.72 [0.63, 0.81]
No	4788	1.00	1.00	1.00
**Surgery**				
Breast-conserving surgery	4255	1.00	1.00	1.00
Mastectomy	2009	0.03 [0.02, 0.03]	1.63 [1.40, 1.91]	0.86 [0.77, 0.97]
No surgery	15	0.15 [0.04, 0.44]	2.28 [0.74, 6.91]	0.86 [0.48, 1.49]
Unknown	17	0.18 [0.06, 0.48]	1.73 [0.66, 4.70]	1.11 [0.66, 1.90]
**Relative weight of shared component**				
Radiotherapy		1.00	—	—
Chemotherapy		0.58 [0.38, 0.83]	1.00	—
Hormonal therapy		0.89 [0.68, 1.17]	1.54 [1.09, 2.28]	1.00
**PPC**		0.9993		

[a]Abbreviations: CrI=Credible interval, N=Number of patients, TRACT=Travel to cancer treatment, IRSAD=Index of relative socio-economic advantage and disadvantage, PPC=Posterior predictive check.

Table 4: Model A2 (2nd shared component for radiotherapy and chemotherapy) for estimated posterior odds ratios of patient characteristics associated with the intended adjuvant therapies, and relative weights between therapies.

Factors	N	Median posterior odds ratios [95% CrI[a]]		
		**Radiotherapy**	**Chemotherapy**	**Hormonal Therapy**
**Road travelling time (TRACT)**				
< 1 hour	4514	1.00	1.00	1.00
1− < 2 hours	502	0.87 [0.55, 1.38]	0.76 [0.47, 1.21]	0.93 [0.67, 1.29]
2− < 4 hours	750	0.65 [0.41, 1.04]	0.92 [0.59, 1.47]	0.98 [0.71, 1.34]
4− < 6 hours	317	0.41 [0.23, 0.75]	0.44 [0.24, 0.81]	0.75 [0.50, 1.09]
6 or more hours	274	0.42 [0.24, 0.77]	0.60 [0.34, 1.11]	0.72 [0.48, 1.05]
**Socio-economic status (IRSAD)**				
Quintile 1 Most disadvantaged	817	0.96 [0.65, 1.39]	0.72 [0.49, 1.06]	1.01 [0.78, 1.33]
Quintile 2	1458	0.81 [0.59, 1.13]	0.85 [0.62, 1.19]	0.92 [0.72, 1.16]
Quintile 3	1762	1.04 [0.76, 1.41]	0.91 [0.68, 1.22]	1.01 [0.82, 1.26]
Quintile 4	1539	0.93 [0.69, 1.26]	0.93 [0.70, 1.23]	1.03 [0.83, 1.27]
Quintile 5 Most advantaged	781	1.00	1.00	1.00
**Age at diagnosis (years)**				
40-49	933	1.07 [0.85, 1.33]	1.84 [1.51, 2.24]	0.83 [0.71, 0.98]
50-59	2064	1.00	1.00	1.00
60-69	2074	0.86 [0.72, 1.03]	0.50 [0.42, 0.60]	1.20 [1.05, 1.37]
70-89	1225	0.36 [0.29, 0.44]	0.12 [0.09, 0.16]	1.38 [1.18, 1.63]
**Indigenous Status**				
Non-Indigenous	5467	1.00	1.00	1.00
Indigenous	63	1.68 [0.83, 3.55]	1.17 [0.60, 2.21]	1.33 [0.88, 2.04]
Indigenous Unknown	766	0.86 [0.69, 1.08]	0.40 [0.31, 0.52]	1.06 [0.88, 1.26]
**Marital Status**				
Married	4150	1.00	1.00	1.00
Never Married	305	0.77 [0.56, 1.09]	0.79 [0.56, 1.09]	0.85 [0.67, 1.08]
Widowed/Divorced/Separated	1665	0.83 [0.71, 0.99]	0.95 [0.80, 1.14]	1.05 [0.93, 1.19]
Marital Unknown	176	0.92 [0.59, 1.46]	1.06 [0.62, 1.75]	0.91 [0.67, 1.24]
**Tumour Stage**				
Localised (Stage I)	4081	1.00	1.00	1.00
Advanced (Stage II, III, IV)	2139	2.44 [2.04, 2.91]	11.23 [9.62, 13.13]	0.87 [0.77, 0.97]
Stage Unknown	76	0.59 [0.32, 1.09]	2.83 [1.43, 5.34]	1.24 [0.84, 1.88]
**Occupation**				
Blue collar	245	1.70 [1.18, 2.48]	1.03 [0.72, 1.47]	0.98 [0.76, 1.27]
White collar	907	1.49 [1.19, 1.88]	1.18 [0.95, 1.47]	1.11 [0.94, 1.30]
Professional	1061	1.50 [1.22, 1.86]	1.38 [1.12, 1.70]	1.09 [0.94, 1.28]
Not in the labour force	2631	1.00	1.00	1.00
Unknown	1452	1.29 [1.06, 1.56]	1.04 [0.84, 1.28]	1.27 [1.09, 1.46]
**Invasive tumour type**				
Invasive Ductal	4074	1.00	1.00	1.00
Tubular	167	0.48 [0.33, 0.71]	0.27 [0.12, 0.55]	0.91 [0.68, 1.22]
Lobular Classical	506	0.96 [0.75, 1.23]	0.73 [0.56, 0.94]	1.72 [1.41, 2.12]
Other	434	0.74 [0.56, 0.97]	0.76 [0.56, 1.04]	0.94 [0.78, 1.16]
Unknown	1115	0.81 [0.67, 0.98]	0.69 [0.57, 0.84]	0.70 [0.61, 0.80]
**First screen diagnosed**				
Yes	1508	0.81 [0.68, 0.96]	0.79 [0.67, 0.93]	0.72 [0.63, 0.81]
No	4788	1.00	1.00	1.00
**Surgery**				
Breast-conserving surgery	4255	1.00	1.00	1.00
Mastectomy	2009	0.03 [0.02, 0.03]	1.63 [1.40, 1.91]	0.86 [0.77, 0.97]
No surgery	15	0.14 [0.04, 0.44]	2.26 [0.73, 6.76]	0.85 [0.49, 1.48]
Unknown	17	0.18 [0.07, 0.46]	1.73 [0.65, 4.73]	1.11 [0.65, 1.90]
**Relative weight of shared component 1**				
Radiotherapy		1.00	—	—
Chemotherapy		0.57 [0.35, 0.85]	1.00	—
Hormonal therapy		0.88 [0.68, 1.15]	1.54 [1.04, 2.52]	1.00
**Relative weight of shared component 2**				
Chemotherapy		0.82 [0.21, 3.77]	1.00	—
**PPC**		0.9989		

[a]Abbreviations: CrI=Credible interval, N=Number of patients, TRACT=Travel to cancer treatment, IRSAD=Index of relative socio-economic advantage and disadvantage, PPC=Posterior predictive check.

Table 5: Model A3 (2nd shared component for radiotherapy and hormonal therapy) for estimated posterior odds ratios of patient characteristics associated with the intended adjuvant therapies, and relative weights between therapies.

Factors	N	Median posterior odds ratios [95% CrI[a]]		
		**Radiotherapy**	**Chemotherapy**	**Hormonal Therapy**
**Road travelling time (TRACT)**				
$<$ 1 hour	4514	1.00	1.00	1.00
1– $<$ 2 hours	502	0.86 [0.55, 1.37]	0.72 [0.45, 1.18]	0.94 [0.67, 1.31]
2– $<$ 4 hours	750	0.66 [0.41, 1.05]	0.88 [0.56, 1.43]	0.98 [0.71, 1.35]
4– $<$ 6 hours	317	0.41 [0.23, 0.74]	0.42 [0.23, 0.77]	0.74 [0.49, 1.11]
6 or more hours	274	0.41 [0.24, 0.73]	0.57 [0.31, 1.02]	0.72 [0.48, 1.06]
**Socio-economic status (IRSAD)**				
Quintile 1 Most disadvantaged	817	0.97 [0.67, 1.40]	0.72 [0.50, 1.04]	1.01 [0.77, 1.33]
Quintile 2	1458	0.82 [0.59, 1.16]	0.85 [0.62, 1.18]	0.91 [0.72, 1.17]
Quintile 3	1762	1.05 [0.79, 1.43]	0.90 [0.68, 1.21]	1.01 [0.81, 1.26]
Quintile 4	1539	0.94 [0.70, 1.25]	0.92 [0.70, 1.21]	1.04 [0.84, 1.28]
Quintile 5 Most advantaged	781	1.00	1.00	1.00
**Age at diagnosis (years)**				
40-49	933	1.07 [0.86, 1.36]	1.85 [1.51, 2.25]	0.83 [0.71, 0.98]
50-59	2064	1.00	1.00	1.00
60-69	2074	0.86 [0.72, 1.03]	0.50 [0.42, 0.60]	1.20 [1.05, 1.37]
70-89	1225	0.36 [0.29, 0.45]	0.12 [0.09, 0.16]	1.39 [1.18, 1.63]
**Indigenous Status**				
Non-Indigenous	5467	1.00	1.00	1.00
Indigenous	63	1.69 [0.84, 3.45]	1.14 [0.60, 2.11]	1.32 [0.88, 2.07]
Indigenous Unknown	766	0.87 [0.70, 1.09]	0.40 [0.30, 0.53]	1.06 [0.89, 1.26]
**Marital Status**				
Married	4150	1.00	1.00	1.00
Never Married	305	0.78 [0.56, 1.09]	0.79 [0.57, 1.10]	0.85 [0.68, 1.08]
Widowed/Divorced/Separated	1665	0.83 [0.71, 0.98]	0.95 [0.80, 1.14]	1.05 [0.93, 1.19]
Marital Unknown	176	0.91 [0.59, 1.45]	1.06 [0.62, 1.77]	0.91 [0.67, 1.24]
**Tumour Stage**				
Localised (Stage I)	4081	1.00	1.00	1.00
Advanced (Stage II, III, IV)	2139	2.44 [2.05, 2.90]	11.19 [9.62, 13.07]	0.86 [0.77, 0.97]
Stage Unknown	76	0.58 [0.32, 1.10]	2.87 [1.46, 5.44]	1.24 [0.83, 1.87]
**Occupation**				
Blue collar	245	1.71 [1.18, 2.49]	1.03 [0.73, 1.48]	0.98 [0.75, 1.26]
White collar	907	1.49 [1.19, 1.88]	1.18 [0.95, 1.46]	1.11 [0.95, 1.31]
Professional	1061	1.50 [1.22, 1.86]	1.38 [1.13, 1.70]	1.09 [0.93, 1.28]
Not in the labour force	2631	1.00	1.00	1.00
Unknown	1452	1.29 [1.06, 1.56]	1.04 [0.85, 1.28]	1.27 [1.09, 1.46]
**Invasive tumour type**				
Invasive Ductal	4074	1.00	1.00	1.00
Tubular	167	0.48 [0.33, 0.72]	0.27 [0.12, 0.55]	0.90 [0.68, 1.21]
Lobular Classical	506	0.95 [0.75, 1.22]	0.73 [0.56, 0.94]	1.72 [1.39, 2.12]
Other	434	0.74 [0.56, 0.96]	0.77 [0.56, 1.02]	0.94 [0.77, 1.15]
Unknown	1115	0.81 [0.67, 0.98]	0.69 [0.57, 0.84]	0.70 [0.61, 0.81]
**First screen diagnosed**				
Yes	1508	0.81 [0.68, 0.97]	0.79 [0.67, 0.93]	0.72 [0.63, 0.82]
No	4788	1.00	1.00	1.00
**Surgery**				
Breast-conserving surgery	4255	1.00	1.00	1.00
Mastectomy	2009	0.03 [0.02, 0.03]	1.63 [1.39, 1.90]	0.86 [0.76, 0.97]
No surgery	15	0.15 [0.04, 0.45]	2.24 [0.76, 7.24]	0.86 [0.48, 1.47]
Unknown	17	0.18 [0.07, 0.48]	1.72 [0.65, 4.54]	1.11 [0.66, 1.89]
**Relative weight of shared component 1**				
Radiotherapy		1.00	—	—
Chemotherapy		0.74 [0.40, 1.71]	1.00	—
Hormonal therapy		0.87 [0.61, 1.26]	1.19 [0.54, 2.09]	1.00
**Relative weight of shared component 2**				
Hormonal Therapy		1.01 [0.32, 2.83]	—	1.00
**PPC**		0.9990		

[a]Abbreviations: CrI=Credible interval, N=Number of patients, TRACT=Travel to cancer treatment, IRSAD=Index of relative socio-economic advantage and disadvantage, PPC=Posterior predictive check.

Table 6: Model A4 (2nd shared component for chemotherapy and hormonal therapy) for estimated posterior odds ratios of patient characteristics associated with the intended adjuvant therapies, and relative weights between therapies.

Factors	N	Median posterior odds ratios [95% CrI[a]]		
		**Radiotherapy**	**Chemotherapy**	**Hormonal Therapy**
**Road travelling time (TRACT)**				
$<$ 1 hour	4514	1.00	1.00	1.00
1$-$ $<$ 2 hours	502	0.86 [0.54, 1.39]	0.73 [0.46, 1.20]	0.94 [0.67, 1.32]
2$-$ $<$ 4 hours	750	0.66 [0.41, 1.06]	0.89 [0.56, 1.44]	0.98 [0.71, 1.35]
4$-$ $<$ 6 hours	317	0.42 [0.23, 0.77]	0.43 [0.23, 0.80]	0.74 [0.50, 1.07]
6 or more hours	274	0.43 [0.23, 0.76]	0.57 [0.33, 1.05]	0.71 [0.48, 1.03]
**Socio-economic status (IRSAD)**				
Quintile 1 Most disadvantaged	817	0.97 [0.67, 1.40]	0.72 [0.49, 1.04]	1.01 [0.77, 1.30]
Quintile 2	1458	0.83 [0.60, 1.15]	0.85 [0.61, 1.15]	0.91 [0.72, 1.16]
Quintile 3	1762	1.06 [0.79, 1.43]	0.90 [0.67, 1.20]	1.01 [0.81, 1.25]
Quintile 4	1539	0.94 [0.70, 1.24]	0.92 [0.69, 1.21]	1.03 [0.84, 1.26]
Quintile 5 Most advantaged	781	1.00	1.00	1.00
**Age at diagnosis (years)**				
40-49	933	1.07 [0.85, 1.33]	1.84 [1.51, 2.26]	0.83 [0.71, 0.98]
50-59	2064	1.00	1.00	1.00
60-69	2074	0.86 [0.72, 1.03]	0.50 [0.42, 0.61]	1.20 [1.05, 1.37]
70-89	1225	0.36 [0.29, 0.45]	0.12 [0.09, 0.16]	1.38 [1.18, 1.63]
**Indigenous Status**				
Non-Indigenous	5467	1.00	1.00	1.00
Indigenous	63	1.69 [0.80, 3.47]	1.15 [0.61, 2.15]	1.32 [0.87, 2.05]
Indigenous Unknown	766	0.87 [0.69, 1.09]	0.40 [0.30, 0.53]	1.05 [0.89, 1.25]
**Marital Status**				
Married	4150	1.00	1.00	1.00
Never Married	305	0.78 [0.56, 1.09]	0.79 [0.57, 1.11]	0.85 [0.67, 1.08]
Widowed/Divorced/Separated	1665	0.83 [0.71, 0.98]	0.95 [0.80, 1.14]	1.05 [0.93, 1.19]
Marital Unknown	176	0.92 [0.59, 1.43]	1.06 [0.62, 1.78]	0.91 [0.67, 1.24]
**Tumour Stage**				
Localised (Stage I)	4081	1.00	1.00	1.00
Advanced (Stage II, III, IV)	2139	2.43 [2.05, 2.90]	11.25 [9.60, 13.13]	0.87 [0.77, 0.98]
Stage Unknown	76	0.58 [0.32, 1.07]	2.84 [1.45, 5.36]	1.24 [0.83, 1.88]
**Occupation**				
Blue collar	245	1.70 [1.18, 2.50]	1.03 [0.71, 1.46]	0.98 [0.76, 1.27]
White collar	907	1.49 [1.19, 1.87]	1.18 [0.95, 1.47]	1.11 [0.94, 1.31]
Professional	1061	1.50 [1.21, 1.86]	1.39 [1.13, 1.70]	1.09 [0.94, 1.27]
Not in the labour force	2631	1.00	1.00	1.00
Unknown	1452	1.29 [1.07, 1.55]	1.04 [0.85, 1.28]	1.27 [1.09, 1.47]
**Invasive tumour type**				
Invasive Ductal	4074	1.00	1.00	1.00
Tubular	167	0.48 [0.33, 0.70]	0.27 [0.12, 0.55]	0.91 [0.66, 1.23]
Lobular Classical	506	0.95 [0.74, 1.23]	0.73 [0.56, 0.94]	1.72 [1.41, 2.13]
Other	434	0.73 [0.56, 0.96]	0.76 [0.57, 1.03]	0.94 [0.77, 1.16]
Unknown	1115	0.81 [0.67, 0.98]	0.69 [0.57, 0.84]	0.70 [0.61, 0.80]
**First screen diagnosed**				
Yes	1508	0.81 [0.68, 0.96]	0.79 [0.66, 0.93]	0.72 [0.63, 0.81]
No	4788	1.00	1.00	1.00
**Surgery**				
Breast-conserving surgery	4255	1.00	1.00	1.00
Mastectomy	2009	0.03 [0.02, 0.03]	1.63 [1.39, 1.91]	0.86 [0.76, 0.97]
No surgery	15	0.15 [0.04, 0.43]	2.26 [0.76, 6.94]	0.86 [0.48, 1.46]
Unknown	17	0.18 [0.07, 0.47]	1.71 [0.63, 4.68]	1.10 [0.66, 1.92]
**Relative weight of shared component 1**				
Radiotherapy		1.00	—	—
Chemotherapy		0.54 [0.31, 0.82]	1.00	—
Hormonal therapy		0.85 [0.62, 1.14]	1.58 [1.02, 2.65]	1.00
**Relative weight of shared component 2**				
Hormonal Therapy		—	1.22 [0.28, 5.45]	1.00
**PPC**		0.9992		

[a]Abbreviations: CrI=Credible interval, N=Number of patients, TRACT=Travel to cancer treatment, IRSAD=Index of relative socio-economic advantage and disadvantage, PPC=Posterior predictive check.

Table 7: Model A5 (without spatially unstructured random effect 'u') for estimated posterior odds ratios of patient characteristics associated with the intended adjuvant therapies, and relative weights between therapies.

Factors	N	Median posterior odds ratios [95% CrI[a]]		
		**Radiotherapy**	**Chemotherapy**	**Hormonal Therapy**
**Road travelling time (TRACT)**				
< 1 hour	4514	1.00	1.00	1.00
1− < 2 hours	502	0.91 [0.57, 1.44]	0.76 [0.48, 1.19]	0.94 [0.68, 1.30]
2− < 4 hours	750	0.67 [0.43, 1.09]	0.89 [0.57, 1.41]	0.96 [0.70, 1.33]
4− < 6 hours	317	0.44 [0.25, 0.77]	0.45 [0.25, 0.81]	0.75 [0.51, 1.08]
6 or more hours	274	0.46 [0.27, 0.81]	0.59 [0.34, 1.06]	0.72 [0.49, 1.03]
**Socio-economic status (IRSAD)**				
Quintile 1 Most disadvantaged	817	0.97 [0.68, 1.38]	0.75 [0.52, 1.06]	1.01 [0.78, 1.30]
Quintile 2	1458	0.81 [0.60, 1.10]	0.88 [0.65, 1.20]	0.92 [0.73, 1.14]
Quintile 3	1762	1.04 [0.79, 1.40]	0.91 [0.70, 1.20]	1.00 [0.82, 1.22]
Quintile 4	1539	0.93 [0.71, 1.22]	0.93 [0.71, 1.22]	1.03 [0.85, 1.25]
Quintile 5 Most advantaged	781	1.00	1.00	1.00
**Age at diagnosis (years)**				
40-49	933	1.07 [0.86, 1.34]	1.83 [1.49, 2.23]	0.84 [0.71, 0.98]
50-59	2064	1.00	1.00	1.00
60-69	2074	0.86 [0.72, 1.02]	0.50 [0.42, 0.60]	1.20 [1.05, 1.37]
70-89	1225	0.37 [0.30, 0.45]	0.12 [0.09, 0.16]	1.38 [1.17, 1.62]
**Indigenous Status**				
Non-Indigenous	5467	1.00	1.00	1.00
Indigenous	63	1.67 [0.82, 3.47]	1.18 [0.63, 2.20]	1.32 [0.87, 2.02]
Indigenous Unknown	766	0.87 [0.70, 1.09]	0.40 [0.30, 0.53]	1.06 [0.89, 1.26]
**Marital Status**				
Married	4150	1.00	1.00	1.00
Never Married	305	0.78 [0.57, 1.08]	0.79 [0.57, 1.08]	0.85 [0.67, 1.09]
Widowed/Divorced/Separated	1665	0.83 [0.71, 0.98]	0.95 [0.80, 1.14]	1.04 [0.92, 1.18]
Marital Unknown	176	0.91 [0.58, 1.43]	1.06 [0.62, 1.77]	0.91 [0.67, 1.24]
**Tumour Stage**				
Localised (Stage I)	4081	1.00	1.00	1.00
Advanced (Stage II, III, IV)	2139	2.41 [2.03, 2.88]	10.98 [9.43, 12.83]	0.87 [0.77, 0.97]
Stage Unknown	76	0.59 [0.32, 1.08]	2.83 [1.40, 5.32]	1.22 [0.82, 1.82]
**Occupation**				
Blue collar	245	1.70 [1.17, 2.47]	1.03 [0.71, 1.45]	0.98 [0.76, 1.27]
White collar	907	1.49 [1.18, 1.85]	1.18 [0.95, 1.46]	1.11 [0.94, 1.30]
Professional	1061	1.50 [1.22, 1.86]	1.38 [1.13, 1.70]	1.09 [0.93, 1.28]
Not in the labour force	2631	1.00	1.00	1.00
Unknown	1452	1.29 [1.07, 1.55]	1.05 [0.85, 1.28]	1.26 [1.09, 1.46]
**Invasive tumour type**				
Invasive Ductal	4074	1.00	1.00	1.00
Tubular	167	0.49 [0.34, 0.71]	0.27 [0.12, 0.54]	0.91 [0.68, 1.22]
Lobular Classical	506	0.95 [0.75, 1.22]	0.73 [0.56, 0.94]	1.71 [1.40, 2.11]
Other	434	0.74 [0.57, 0.97]	0.76 [0.57, 1.02]	0.95 [0.77, 1.16]
Unknown	1115	0.82 [0.67, 0.98]	0.69 [0.57, 0.84]	0.70 [0.61, 0.80]
**First screen diagnosed**				
Yes	1508	0.81 [0.69, 0.96]	0.79 [0.67, 0.94]	0.72 [0.63, 0.82]
No	4788	1.00	1.00	1.00
**Surgery**				
Breast-conserving surgery	4255	1.00	1.00	1.00
Mastectomy	2009	0.03 [0.02, 0.04]	1.63 [1.39, 1.91]	0.86 [0.77, 0.97]
No surgery	15	0.15 [0.04, 0.43]	2.23 [0.75, 6.71]	0.87 [0.49, 1.49]
Unknown	17	0.18 [0.07, 0.48]	1.65 [0.59, 4.51]	1.09 [0.65, 1.85]
**Relative weight of shared component**				
Radiotherapy		1.00	—	—
Chemotherapy		0.54 [0.33, 0.79]	1.00	—
Hormonal therapy		0.85 [0.65, 1.10]	1.57 [1.08, 2.53]	1.00
**PPC**		0.9992		

[a]Abbreviations: CrI=Credible interval, N=Number of patients, TRACT=Travel to cancer treatment, IRSAD=Index of relative socio-economic advantage and disadvantage, PPC=Posterior predictive check.

Table 8: Model A6 (without spatially structured random effect 's') for estimated posterior odds ratios of patient characteristics associated with the intended adjuvant therapies, and relative weights between therapies.

Factors	N	Median posterior odds ratios [95% CrI[a]]		
		**Radiotherapy**	**Chemotherapy**	**Hormonal Therapy**
**Road travelling time (TRACT)**				
< 1 hour	4514	1.00	1.00	1.00
1− < 2 hours	502	0.84 [0.55, 1.30]	0.57 [0.39, 0.85]	0.96 [0.68, 1.34]
2− < 4 hours	750	0.62 [0.43, 0.89]	0.74 [0.54, 1.01]	0.99 [0.73, 1.35]
4− < 6 hours	317	0.39 [0.23, 0.65]	0.35 [0.22, 0.56]	0.77 [0.51, 1.11]
6 or more hours	274	0.36 [0.23, 0.61]	0.46 [0.28, 0.72]	0.75 [0.51, 1.08]
**Socio-economic status (IRSAD)**				
Quintile 1 Most disadvantaged	817	0.89 [0.62, 1.27]	0.67 [0.47, 0.95]	1.04 [0.80, 1.35]
Quintile 2	1458	0.76 [0.55, 1.04]	0.79 [0.58, 1.07]	0.95 [0.75, 1.20]
Quintile 3	1762	0.94 [0.72, 1.30]	0.83 [0.64, 1.09]	1.06 [0.85, 1.30]
Quintile 4	1539	0.88 [0.65, 1.17]	0.86 [0.66, 1.12]	1.07 [0.87, 1.32]
Quintile 5 Most advantaged	781	1.00	1.00	1.00
**Age at diagnosis (years)**				
40-49	933	1.07 [0.85, 1.35]	1.85 [1.52, 2.25]	0.83 [0.71, 0.97]
50-59	2064	1.00	1.00	1.00
60-69	2074	0.86 [0.72, 1.02]	0.51 [0.42, 0.60]	1.20 [1.05, 1.37]
70-89	1225	0.36 [0.29, 0.44]	0.13 [0.10, 0.17]	1.38 [1.18, 1.61]
**Indigenous Status**				
Non-Indigenous	5467	1.00	1.00	1.00
Indigenous	63	1.66 [0.81, 3.43]	1.10 [0.58, 2.05]	1.35 [0.90, 2.09]
Indigenous Unknown	766	0.86 [0.69, 1.08]	0.40 [0.30, 0.53]	1.06 [0.89, 1.26]
**Marital Status**				
Married	4150	1.00	1.00	1.00
Never Married	305	0.79 [0.57, 1.10]	0.80 [0.57, 1.13]	0.85 [0.67, 1.07]
Widowed/Divorced/Separated	1665	0.84 [0.71, 0.99]	0.96 [0.81, 1.14]	1.04 [0.92, 1.19]
Marital Unknown	176	0.92 [0.59, 1.42]	1.06 [0.63, 1.75]	0.91 [0.67, 1.23]
**Tumour Stage**				
Localised (Stage I)	4081	1.00	1.00	1.00
Advanced (Stage II, III, IV)	2139	2.43 [2.04, 2.90]	11.09 [9.48, 12.92]	0.87 [0.77, 0.97]
Stage Unknown	76	0.59 [0.32, 1.09]	2.75 [1.39, 5.16]	1.23 [0.84, 1.88]
**Occupation**				
Blue collar	245	1.71 [1.19, 2.48]	1.03 [0.71, 1.47]	0.98 [0.76, 1.28]
White collar	907	1.49 [1.19, 1.86]	1.20 [0.97, 1.48]	1.11 [0.95, 1.30]
Professional	1061	1.48 [1.20, 1.84]	1.38 [1.13, 1.69]	1.10 [0.94, 1.28]
Not in the labour force	2631	1.00	1.00	1.00
Unknown	1452	1.26 [1.04, 1.52]	1.05 [0.85, 1.28]	1.27 [1.10, 1.47]
**Invasive tumour type**				
Invasive Ductal	4074	1.00	1.00	1.00
Tubular	167	0.48 [0.33, 0.70]	0.27 [0.12, 0.55]	0.91 [0.68, 1.22]
Lobular Classical	506	0.96 [0.74, 1.23]	0.72 [0.56, 0.94]	1.72 [1.42, 2.12]
Other	434	0.74 [0.57, 0.96]	0.78 [0.58, 1.04]	0.94 [0.77, 1.15]
Unknown	1115	0.81 [0.68, 0.98]	0.69 [0.57, 0.85]	0.70 [0.61, 0.81]
**First screen diagnosed**				
Yes	1508	0.81 [0.69, 0.96]	0.79 [0.67, 0.93]	0.72 [0.63, 0.82]
No	4788	1.00	1.00	1.00
**Surgery**				
Breast-conserving surgery	4255	1.00	1.00	1.00
Mastectomy	2009	0.03 [0.02, 0.03]	1.62 [1.38, 1.90]	0.87 [0.77, 0.98]
No surgery	15	0.14 [0.04, 0.42]	2.40 [0.78, 7.52]	0.84 [0.48, 1.41]
Unknown	17	0.18 [0.07, 0.48]	1.69 [0.64, 4.56]	1.11 [0.66, 1.91]
**Relative weight of shared component**				
Radiotherapy		1.00	—	—
Chemotherapy		0.44 [0.28, 0.64]	1.00	—
Hormonal therapy		0.98 [0.80, 1.22]	2.25 [1.55, 3.48]	1.00
**PPC**		0.9990		

[a]Abbreviations: CrI=Credible interval, N=Number of patients, TRACT=Travel to cancer treatment, IRSAD=Index of relative socio-economic advantage and disadvantage, PPC=Posterior predictive check.

Table 9: Model A7 (flat priors for intercept and regression coefficients) for estimated posterior odds ratios of patient characteristics associated with the intended adjuvant therapies, and relative weights between therapies.

Factors	N	Median posterior odds ratios [95% CrI[a]]		
		Radiotherapy	Chemotherapy	Hormonal Therapy
**Road travelling time (TRACT)**				
< 1 hour	4514	1.00	1.00	1.00
1− < 2 hours	502	0.60 [0.36, 1.01]	0.60 [0.36, 1.01]	0.64 [0.40, 1.00]
2− < 4 hours	750	0.37 [0.21, 0.65]	0.64 [0.39, 1.12]	0.55 [0.32, 0.92]
4− < 6 hours	317	0.19 [0.10, 0.38]	0.26 [0.13, 0.53]	0.33 [0.18, 0.64]
6 or more hours	274	0.20 [0.11, 0.40]	0.38 [0.20, 0.76]	0.33 [0.18, 0.64]
**Socio-economic status (IRSAD)**				
Quintile 1 Most disadvantaged	817	0.97 [0.66, 1.41]	0.72 [0.49, 1.06]	1.04 [0.76, 1.42]
Quintile 2	1458	0.80 [0.57, 1.10]	0.84 [0.60, 1.18]	0.90 [0.69, 1.19]
Quintile 3	1762	1.02 [0.75, 1.38]	0.89 [0.66, 1.20]	1.00 [0.78, 1.28]
Quintile 4	1539	0.91 [0.67, 1.21]	0.91 [0.68, 1.22]	1.02 [0.81, 1.29]
Quintile 5 Most advantaged	781	1.00	1.00	1.00
**Age at diagnosis (years)**				
40-49	933	1.07 [0.85, 1.35]	1.85 [1.51, 2.27]	0.83 [0.70, 0.98]
50-59	2064	1.00	1.00	1.00
60-69	2074	0.85 [0.71, 1.02]	0.49 [0.41, 0.59]	1.22 [1.06, 1.40]
70-89	1225	0.35 [0.28, 0.43]	0.11 [0.09, 0.15]	1.43 [1.21, 1.69]
**Indigenous Status**				
Non-Indigenous	5467	1.00	1.00	1.00
Indigenous	63	1.86 [0.86, 4.17]	1.19 [0.58, 2.38]	1.72 [0.96, 3.16]
Indigenous Unknown	766	0.86 [0.69, 1.08]	0.38 [0.29, 0.51]	1.07 [0.89, 1.28]
**Marital Status**				
Married	4150	1.00	1.00	1.00
Never Married	305	0.77 [0.55, 1.10]	0.77 [0.55, 1.08]	0.83 [0.64, 1.07]
Widowed/Divorced/Separated	1665	0.83 [0.71, 0.98]	0.96 [0.80, 1.14]	1.04 [0.91, 1.18]
Marital Unknown	176	0.92 [0.57, 1.48]	1.06 [0.59, 1.83]	0.86 [0.60, 1.26]
**Tumour Stage**				
Localised (Stage I)	4081	1.00	1.00	1.00
Advanced (Stage II, III, IV)	2139	2.49 [2.09, 2.98]	11.64 [9.98, 13.61]	0.86 [0.77, 0.97]
Stage Unknown	76	0.67 [0.33, 1.33]	3.18 [1.49, 6.55]	1.70 [0.96, 3.02]
**Occupation**				
Blue collar	245	1.75 [1.20, 2.59]	1.04 [0.72, 1.51]	0.99 [0.74, 1.31]
White collar	907	1.50 [1.19, 1.89]	1.18 [0.95, 1.47]	1.14 [0.95, 1.35]
Professional	1061	1.51 [1.22, 1.87]	1.39 [1.12, 1.73]	1.12 [0.94, 1.31]
Not in the labour force	2631	1.00	1.00	1.00
Unknown	1452	1.30 [1.07, 1.58]	1.05 [0.85, 1.29]	1.29 [1.11, 1.50]
**Invasive tumour type**				
Invasive Ductal	4074	1.00	1.00	1.00
Tubular	167	0.47 [0.31, 0.69]	0.18 [0.06, 0.43]	0.88 [0.64, 1.25]
Lobular Classical	506	0.95 [0.74, 1.25]	0.71 [0.54, 0.93]	1.84 [1.48, 2.28]
Other	434	0.72 [0.55, 0.97]	0.75 [0.56, 1.01]	0.94 [0.75, 1.15]
Unknown	1115	0.81 [0.67, 0.98]	0.68 [0.55, 0.82]	0.68 [0.59, 0.79]
**First screen diagnosed**				
Yes	1508	0.81 [0.69, 0.96]	0.78 [0.65, 0.93]	0.71 [0.62, 0.81]
No	4788	1.00	1.00	1.00
**Surgery**				
Breast-conserving surgery	4255	1.00	1.00	1.00
Mastectomy	2009	0.03 [0.02, 0.03]	1.66 [1.41, 1.94]	0.86 [0.76, 0.97]
No surgery	15	0.04 [0.01, 0.19]	3.61 [0.85, 14.65]	0.41 [0.12, 1.41]
Unknown	17	0.10 [0.03, 0.29]	2.38 [0.69, 7.88]	1.51 [0.52, 4.65]
**Relative weight of shared component**				
Radiotherapy		1.00	—	—
Chemotherapy		0.57 [0.36, 0.86]	1.00	—
Hormonal therapy		0.87 [0.63, 1.13]	1.51 [0.97, 2.39]	1.00
**PPC**			0.9991	

[a]Abbreviations: CrI=Credible interval, N=Number of patients, TRACT=Travel to cancer treatment, IRSAD=Index of relative socio-economic advantage and disadvantage, PPC=Posterior predictive check.
