# Supplementary material for: Geographic variation in the intended choice of adjuvant treatments for women diagnosed with screen-detected breast cancer in Queensland
Source: BMC Public Health. 2015 Dec 2;15:1204. doi: 10.1186/s12889-015-2527-2 (PMC4668608; doi:10.1186/s12889-015-2527-2)
Supplement: Additional file 2 — Supplementary Figures. (ZIP 28672 kb) [file 12889_2015_2527_MOESM2_ESM.zip › 12889_2015_MOSEM1_ESM/1403555084156788_add2.pdf]

Supplementary materials

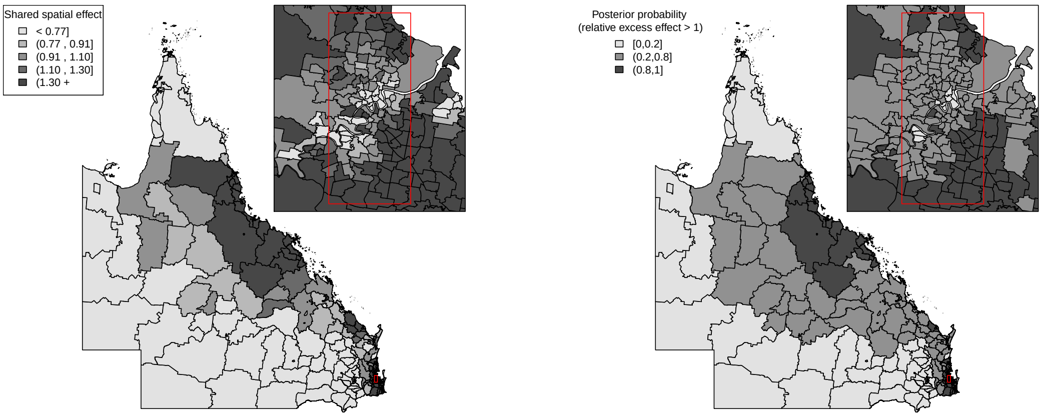

(a) Age group 40-49 (b) Level of uncertainty

Figure 1: Posterior median odds ratio maps for the 40-49 age group shared component effect, 1997–2008.

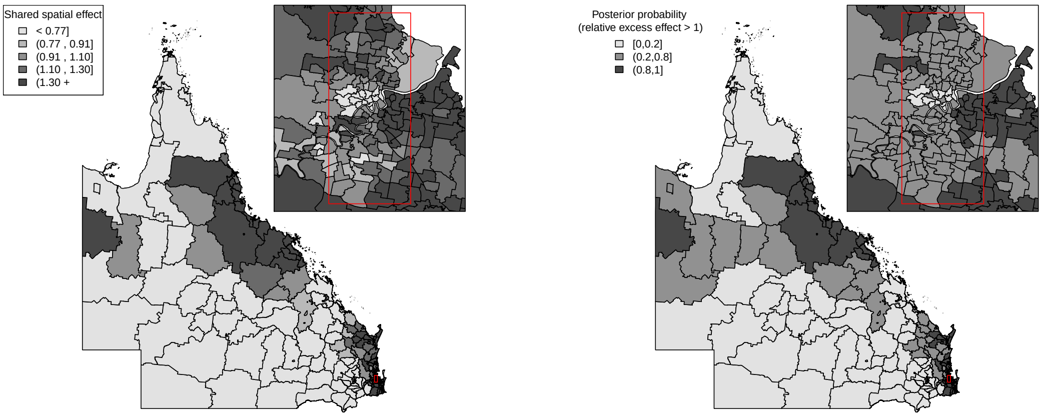

(a) Age group 50-59 (b) Level of uncertainty

Figure 2: Posterior median odds ratio maps for the 50-59 age group shared component effect, 1997–2008.

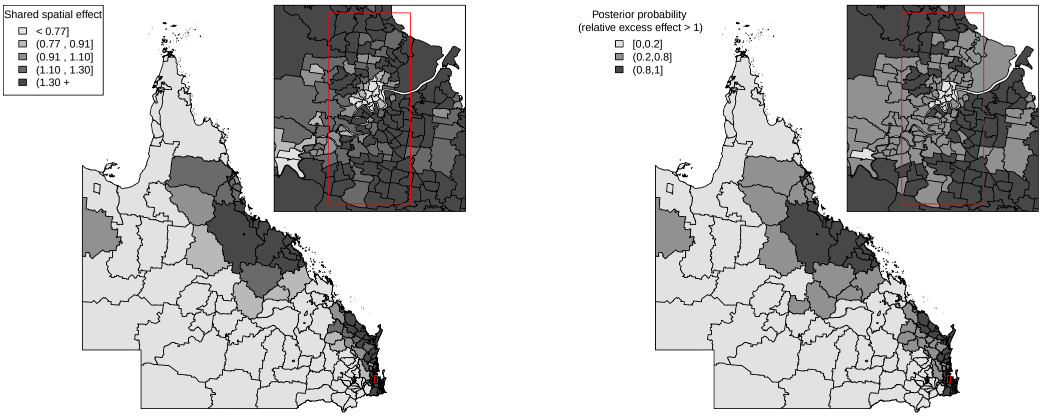

(a) Age group 60-69 (b) Level of uncertainty

Figure 3: Posterior median odds ratio maps for the 60-69 age group shared component effect, 1997–2008.

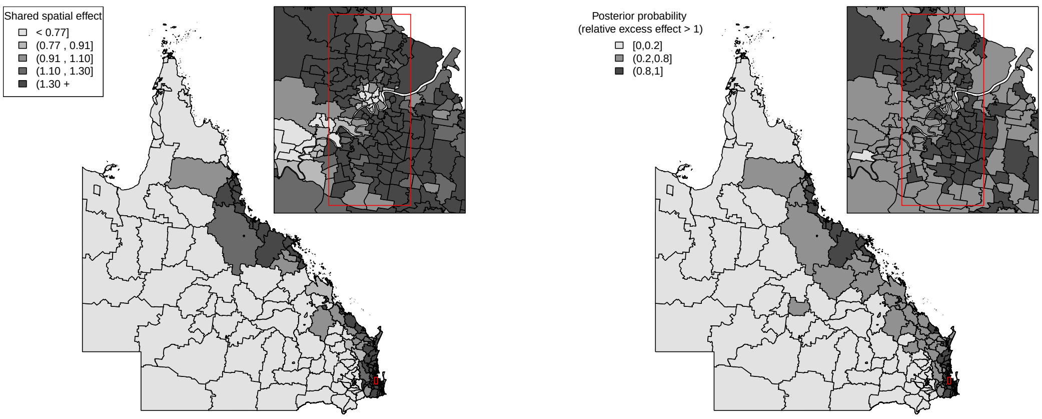

(a) Age group 70-89 (b) Level of uncertainty

Figure 4: Posterior median odds ratio maps for the 70-89 age group shared component effect, 1997–2008.
