# Supplementary material for: Geographic variation in the intended choice of adjuvant treatments for women diagnosed with screen-detected breast cancer in Queensland
Source: BMC Public Health. 2015 Dec 2;15:1204. doi: 10.1186/s12889-015-2527-2 (PMC4668608; doi:10.1186/s12889-015-2527-2)
Supplement: Additional file 2 — Supplementary Figures. (ZIP 28672 kb) [file 12889_2015_2527_MOESM2_ESM.zip › 12889_2015_MOSEM1_ESM/1403555084156788_add3.pdf]

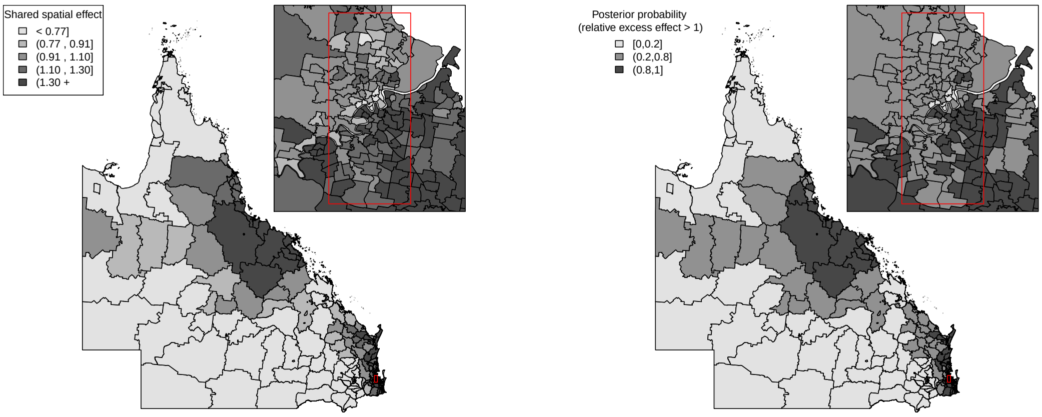

(a) Localised tumour

(b) Level of uncertainty

Figure 5: Posterior median odds ratio maps for the localised tumour shared component effect, 1997–2008.

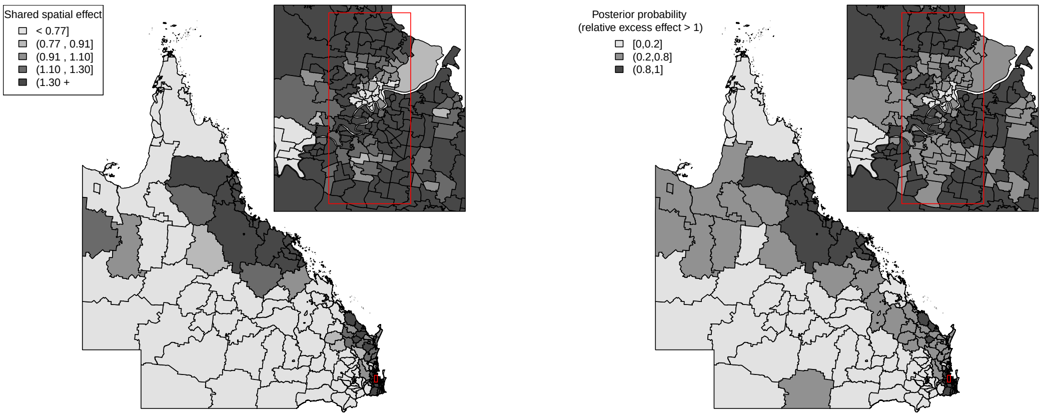

(a) Advanced tumour

(b) Level of uncertainty

Figure 6: Posterior median odds ratio maps for the advanced tumour shared component effect, 1997–2008.

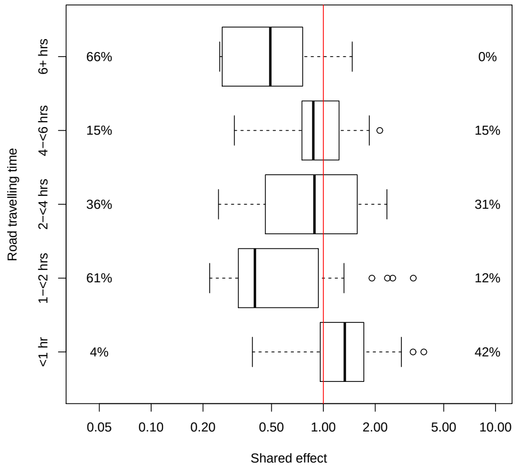

(a) Age group 40-49

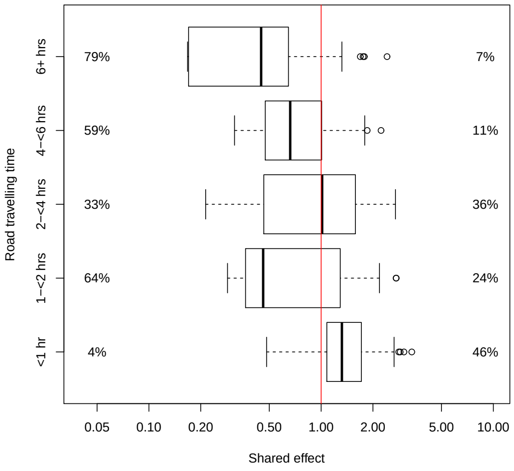

(b) Age group 50-59

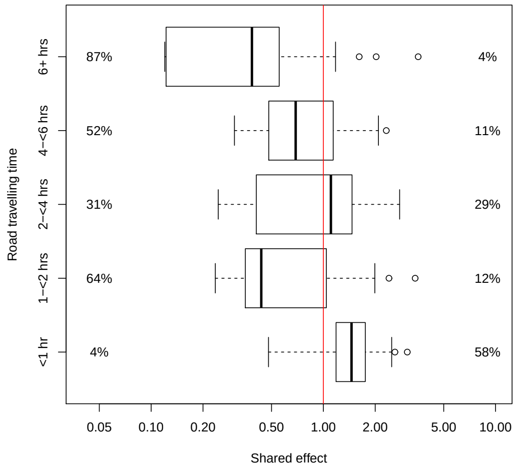

(c) Age group 60-69

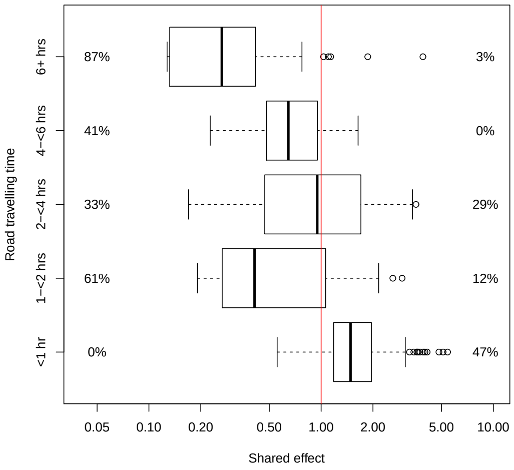

(d) Age group 70-89

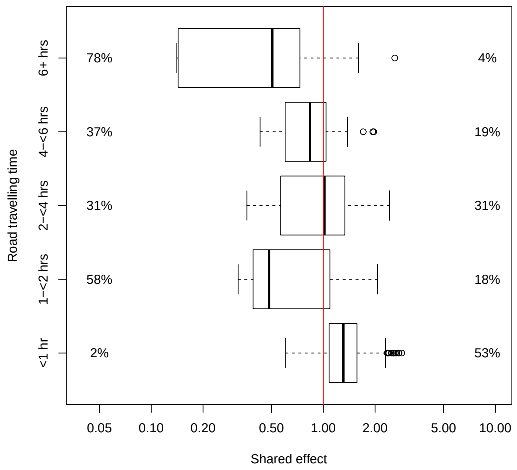

(e) Localised tumour

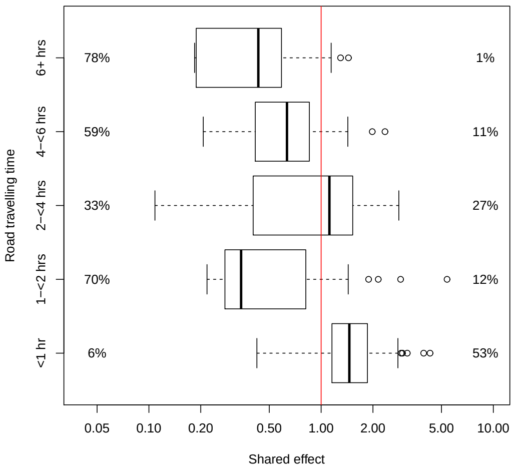

(f) Advanced tumour

Figure 7: Box plots for the median shared component effect by road travelling time for age and tumour stage at diagnosis, 1997–2008.

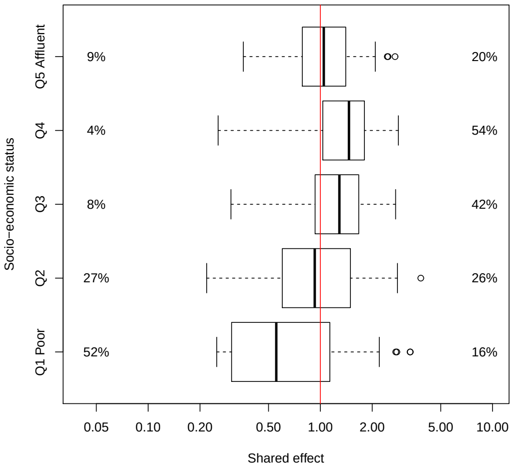

(a) Age group 40-49

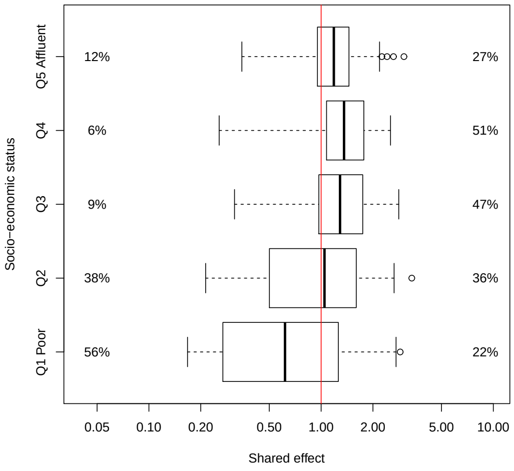

(b) Age group 50-59

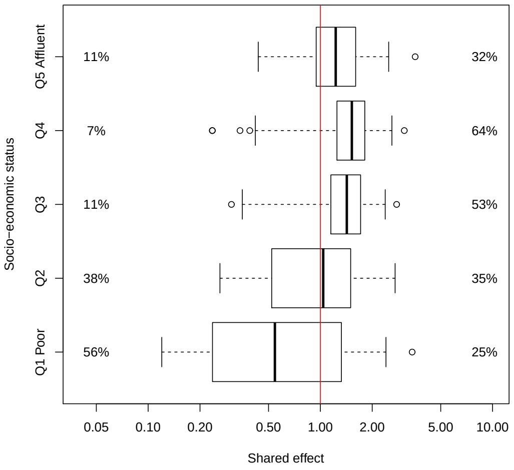

(c) Age group 60-69

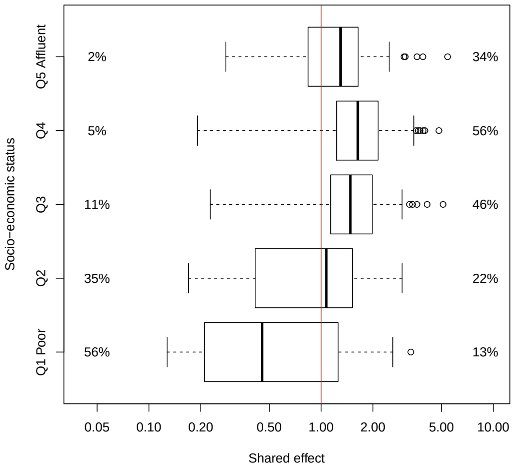

(d) Age group 70-89

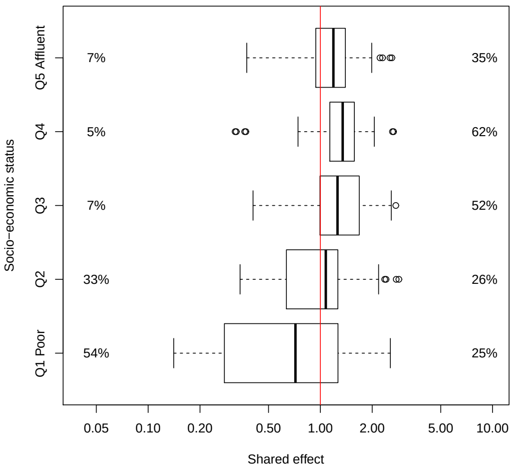

(e) Localised tumour

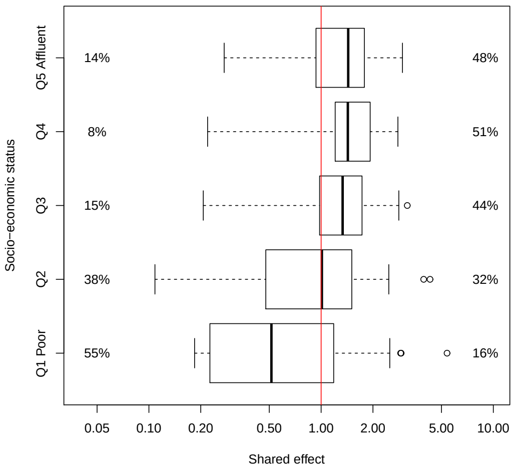

(f) Advanced tumour

Figure 8: Box plots for the median shared component effect by socio-economic status for age and tumour stage at diagnosis, 1997–2008.

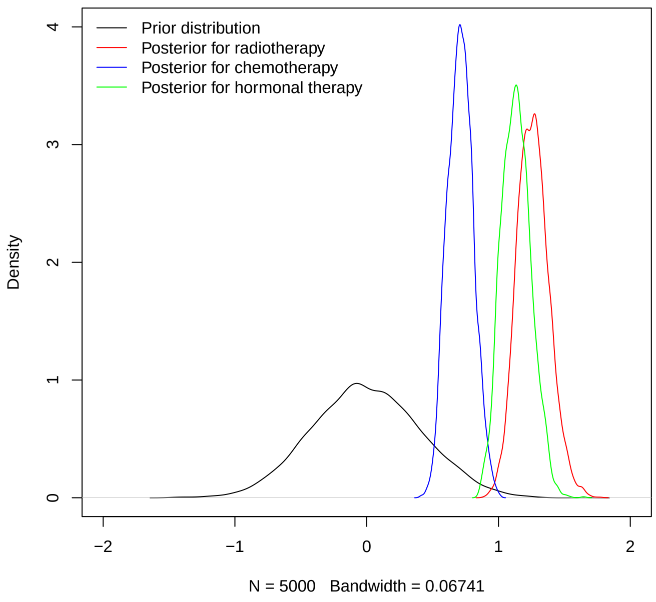

Figure 9: Distribution of shared component weights δ.
